# Morphine for chronic breathlessness (MABEL) in the UK: a health economic evaluation of a multisite, parallel-group, dose titration, double-blind, randomised, placebo-controlled trial

**DOI:** 10.1136/bmjopen-2025-102124

**Published:** 2025-11-04

**Authors:** Marek Jan Atter, Peter Hall, Rachael A Evans, John Norrie, Judith Cohen, Bronwen Williams, Nazia Chaudhuri, Sabrina Bajwah, Irene Higginson, Mark Pearson, David Currow, Gareth Stewart, Marie Fallon, Miriam Johnson

**Affiliations:** 1 Edinburgh Clinical Trials Unit, The University of Edinburgh, Edinburgh, Scotland, UK; 2 Cancer Research Centre, The University of Edinburgh, Edinburgh, Scotland, UK; 3 University of Leicester, Leicester, England, UK; 4 Dentistry and Biomedical Sciences, Queen’s University Belfast, Belfast, Northern Ireland, UK; 5 Hull Health Trials Unit, University of Hull, Hull, England, UK; 6 School of Medicine, Department of Life and Health Sciences, Ulster University, Coleraine, Northern Ireland, UK; 7 Cicely Saunders Institute for Palliative Care, Policy & Rehabilitation, King's College London, London, England, UK; 8 Hull York Medical School, University of Hull, Hull, England, UK; 9 Flinders Ageing Alliance, Flinders University, Bedford Park, South Australia, Australia; 10 NHS Lothian, Edinburgh, Scotland, UK

**Keywords:** Economics, Clinical Trial, PALLIATIVE CARE

## Abstract

**Objectives:**

To compare costs and health consequences and to assess the cost-effectiveness of using low-dose oral long-acting morphine in people with chronic breathlessness.

**Design:**

Within-trial planned cost-consequences and cost-effectiveness analysis of data from a multisite, parallel-group, double-blind, randomised, placebo-controlled trial of low-dose, long-acting morphine.

**Setting:**

11 hospital outpatients across the UK.

**Participants:**

Consenting adults with chronic breathlessness due to long-term cardiorespiratory conditions.

**Intervention:**

5–10 mg two times a day oral long-acting morphine with a blinded laxative for 56 days.

**Primary outcome measures:**

Mean and SD of healthcare resource use (HRU) by trial arm; mean differences and 95% CI of costs between trial arms.

**Secondary outcome measures:**

Mean differences in 28- and 56-day quality-adjusted life years (QALYs based on EuroQol five-dimension five-level score), Short Form-six dimensional scores and ICEpop CAPability-Supportive Care Measure scores; cost-utility of long-acting morphine for chronic breathlessness.

**Results:**

143 participants (75 morphine and 68 placebo) were randomised; 140 (90% power, males 66%, mean age 70.5 (SD 9.4)) formed the modified intention-to-treat population (participants receiving at least one dose of study medication). There were more inpatient and fewer outpatient services used by the morphine group versus the placebo. In the base-case analysis at 56 days, long-acting morphine was associated with similar mean per-patient costs and QALYs. There was an increase of £24 (95% CI −£395 to £552) and 0.002 (95% CI −0.004 to 0.008) QALYs. Hospitalisations were the main driver of cost differences. The corresponding incremental cost-effectiveness ratio was £12 000/QALY, with a probability of cost-effectiveness of 54% at a £20 000 willingness-to-pay threshold. In the scenario analysis that excluded costs of adverse events considered unrelated to long-acting morphine by site investigators and researchers, the probability of cost-effectiveness increased to 73%.

**Conclusion:**

Oral morphine for chronic breathlessness is likely to be a cost-effective intervention provided adverse events are minimised, but the effect on outcome is small and cautious interpretation is warranted.

**Trial registration number:**

ISRCTN87329095.

**STRENGTHS AND LIMITATIONS OF THIS STUDY**

- Comprehensive collection of patient-reported health economic data in a randomised controlled trial, including three different health outcome measures relevant to people living with chronic breathlessness due to medical conditions.
- The parent trial intervention dosing schedule mirrored clinical practice to give a pragmatic indication of cost impacts.
- Limited interpretation of cost-effectiveness analysis due to a likely random imbalance in deaths and expensive adverse events, in a study design not primarily designed to detect differences in economic endpoints.
- Technical challenges in analysing and estimating costs for concomitant medications.

## Introduction

 Chronic breathlessness is a disabling symptom common in cardiorespiratory diseases and cancer.^1 2^ Breathlessness is a global problem, with population prevalence ranging from 9% in high-income countries to 40% in low-income countries such as India, mirroring countries with higher rates of causal diseases, higher smoking rates and greater levels of environmental pollution.^3 4^ Despite the significant adverse impacts of chronic, or persistent, breathlessness on quality of life, physical and psychosocial functioning and healthcare service utilisation, effective management is often neglected.^5–7^

Opioids have shown potential in reducing the perception of breathlessness by modulating central pathways, but clinical trials in ambulant populations have not consistently demonstrated benefits, partly due to short study durations and the lack of measures of exercise endurance. In addition, morphine has a well-known adverse effect profile, including complications which could plausibly increase health service costs, complications from gastrointestinal and neurocognitive side effects and respiratory depression. The health economic implications of chronic breathlessness beyond the specific context of chronic obstructive pulmonary disease (COPD) have not been comprehensively studied. The only published health economic evaluation, to our knowledge, however, found a high likelihood that 4 weeks of long-acting morphine was cost-effective, compared with placebo, in the management of people living with chronic breathlessness due to COPD.^7^

The Morphine And BrEathLessness (MABEL) trial (registration ISRCTN 87329095) aimed to assess the effectiveness, cost-consequences, cost-effectiveness and safety of long-acting low-dose, oral morphine on patient-reported worst breathlessness in people with chronic breathlessness.^8^

The main clinical results of the MABEL trial were published separately.^9^ This paper contains the health economic component of the MABEL trial, which aimed to compare the costs, health outcomes (cost-consequences analysis) and cost-effectiveness of using morphine for patient-reported *worst breathlessness* in people with chronic breathlessness.

## Methods

### Trial overview

Full details of the MABEL trial, its procedures (including inclusion/exclusion criteria) and clinical findings can be found elsewhere; a summary is included here for context.^8 9^ Ethical approval for MABEL was obtained on 3 December 2019 from the North East-Tyne and Wear South Research Ethics Committee (ref: 19/NE/0284).

MABEL was an 11-site, UK-based, multicentre, phase III, parallel-group, double-blind, randomised, placebo-controlled titration trial. Eligible patients were ambulant adults with moderate to severe chronic breathlessness (modified Medical Research Council (mMRC) ≥grade 3 or 4) due to cardiorespiratory disease or cancer, with adequate renal function, who gave informed consent.

A sample size of 126 (63 participants per group) was needed to detect a moderate effect size (90% power and 5% level of significance), increasing to 158 (79 per group) to allow for 20% attrition. In total, 143 participants were randomised (active/morphine arm, n=75; placebo arm, n=68), of which 140 formed the modified intention-to-treat population (active/morphine arm, n=73; placebo arm, n=67). The modified intention-to-treat population included all participants taking at least one dose of study medication.

Participants were randomised to receive either oral long-acting morphine (5 mg two times a day) with docusate laxative for proactive management of expected constipation side effects (100 mg two times a day) or matching placebo capsules. All medication and packaging were identical to maintain blinding. If the worst breathlessness in the past 24 hours measured by a 0–10 numerical rating scale (NRS) had not improved by at least one point at day 7, and side effects were acceptable, the dose could be increased to 10 mg two times a day starting on day 15, allowing time for dispensing. Participants continued on 5 mg two times a day until the increased dose was supplied. Dose escalation was permitted only if side effects were acceptable, defined as no side effects (common terminology criteria for adverse events (CTCAE) grade 0) or mild gastrointestinal effects (CTCAE ≤grade 2) and no neurocognitive symptoms (CTCAE grade 0, or grade 1 if present at baseline), with ongoing monitoring and agreement from both participant and clinician. The morphine used (MST Continus) was encapsulated for blinding. The treatment duration was 56 days.

The primary outcome was the worst breathlessness in the past 24 hours at day 28. Outcome measures pertinent to the health economic evaluation were the Short-Form 12 (SF-12), the EuroQol five-dimension five-level score (EQ-5D-5L), the ICEpop CAPability-Supportive Care Measure (ICECAP-SCM) and the health resource use (HRU) questionnaire measured at baseline, day 28 and day 56.^10–13^ Attribution of serious adverse events to the study drug was assigned by the recruiting site clinician and discussed with the clinical chief investigator (with over 30 years of experience in opioid prescribing).

Given that the MABEL health economic analysis relies on a placebo-controlled trial of an inexpensive drug-based intervention expected to constitute a negligible share of overall treatment costs, most drivers of costs were expected to stem from secondary outcomes (eg, HRU items such as hospitalisations) rather than the drug itself.

### Methods for the economic analysis

The paper follows Consolidated Health Economic Evaluation Reporting Standards guidance for health economic evaluations (summarised in online supplemental table 1).^14^ The methods of calculating costs, health outcomes and cost-effectiveness metrics were stipulated in a Health Economic Analysis Plan (HEAP) approved by the lead economist and chief investigator before the data lock and unblinding (see online supplemental materials).

In line with guidance, all costs and health outcomes were calculated from a National Health Service (NHS) and Personal Social Services (healthcare payer) perspective, which includes the costs of primary and secondary care and community NHS activity in England.^15^

As per the National Institute for Health and Care Excellence (NICE) guidelines, the health outcome of interest is the quality-adjusted life year (QALY), which is an ‘index of survival that is adjusted to account for the patient’s quality of life’ best calculated using the EQ-5D-5L measure.^15 16^

In light of the evolving discussion in the scientific literature about the interpretation of the QALY in end-of-life settings, SF-6D (derived from SF-12) and the capability measure ICECAP-SCM were selected as alternative measures to present alongside EQ-5D-5L results.^10 11^ The SF-6D may offer greater sensitivity than the EQ-5D due to its larger descriptive system, although this is offset by inconsistencies in some of its upper-level dimension coefficients, while ICECAP presents a measure more adapted to the palliative care context with its focus on capability scoring.^10 11^

However, a few items outlined in the HEAP were not implemented in the final analysis, including the societal perspective (eg, productivity loss due to illness) calculations and QALYs derived from non-EQ-5D-5L measures (eg, calculated from SF-6D instead). This was due to delayed project timelines (due to the COVID-19 pandemic) and researcher availability.

### Data collection

The data needed for the health economic analysis were drawn from a combination of trial case report form (CRF) data and a modified version of the UK Cancer Costs Questionnaire collected on day 28 and day 56 after randomisation.^13^ The time horizon used in this paper is therefore 56 days, but selected key results for day 28 are also presented. With a time horizon of under 1 year, no discounting of costs or outcomes was applied.

### Estimating outcomes

QALYs were calculated individually for each participant in the modified intention-to-treat population using two steps. First, Health State Utility Values (HSUVs) were sourced from EQ-5D-5L Patient-Reported Outcome Measure questionnaire data at each time point using a validated mapping function by Hernández Alava *et al*^17^ Second, 28-day and 56-day QALYs were calculated as a function of HSUVs and their corresponding time points using the area under the curve method outlined by Manca *et al.*^18^

The SF-6D was converted into HSUVs using validated ProCore software provided by QualityMetric, while the ICECAP-SCM measure was converted into tariff values for its capability score, which ranges from 0 (no capability) to 1 (full capability), using its published value set.^19 20^ SF-6D and ICECAP-SCM scores were not used in the QALY calculation or the cost-effectiveness analysis.

### Estimating costs

Price weights used to calculate per-patient costs of hospital and community (hospice) resource use are presented in table 1. Medication unit costs, which include morphine as the investigational medicinal product (IMP), laxatives as the non-investigational medicinal product (NIMP) and concomitant medications, were sourced from the British National Formulary (BNF).^21^

**Table 1:** Morphine and breathlessness trial HRU unit costs (reported in 2021/2022 Great British Pounds)

Item	Cost	Ref	Notes
**Hospital health resources**			
Inpatient stay	£401	22	Approximation of a bed day cost, assumed to be half the cost of a short non-elective inpatient stay, defined as 1–2 nights (index: ‘non-elective inpatient—short stay’); this cost is excluded from the scenario analysis and replaced by SAE-specific costs
Emergency assessment	£242	22	Index: ‘Emergency Care’
Hospital consultant/doctor	£224	22	Weighted average for outpatient face-to-face consultant-led ‘Respiratory Medicine Service’ entries
Specialist nurse	£110	22	Index: ‘Specialist Nursing, Asthma and Respiratory Nursing/Liaison, Adult, Face to Face’
Physiotherapist	£100	22	Index: ‘Physiotherapy Service’
Dietician	£105	22	Index: ‘Dietetics Service’
Occupational therapy	£106	22	Index: ‘Occupational Therapy Service’
Social worker	£53	32	No information on average visit duration, so the hourly unit cost for a qualified social worker (adult services) is assumed as a reasonable approximation
Optometry	£138	22	Index: ‘Optometry Service’
CT	£140	22	Weighted average for adult CT scan entries in the ‘Total HRGs’ tab
X-ray	£41	22	Index: ‘Plain Film’ (outpatient)
Ultrasound	£73	22	Weighted average for ultrasound entries in the ‘IMAG’ tab
Osteopathy	£100	22	Index: ‘Physiotherapy Service’ (no distinct osteopathy cost was identified; physiotherapy cost was assumed as an approximation)
Music therapy	£15	22	Index: ‘Music Therapy Service’
Magnetic resonance	£213	22	Weighted average for adult MRI scan entries in “Total HRGs”
Phlebotomy	£5	22	Index: ‘Phlebotomy’
Telehealth service	£9	32	Nurse-led triage (cost per intervention)
Hospital consultant/doctor (phone call)	£49	22	Index: ‘Follow-up, Non Face to Face’
Specialist nurse (phone call)	£70	22	Index: ‘Specialist Nursing, Asthma and Respiratory Nursing/Liaison, Adult, Non Face to Face’
Administrator/secretary (phone call)	£9	32	Assumed to be approximated by: nurse-led triage (cost per intervention)
**Community health resources**			
Hospice Inpatient Stay	£305	32	Inpatient, specialist palliative care (19 years and over), average cost per bed day (19 years and over)
Doctor	£76	22	Index: ‘Follow-up, Adult, Face to Face’
Nurse	£54	22	Index: ‘District Nurse, Adult, Face to Face’
Day hospice	£80	22	Index: ‘Community health services’
Other professional	£253	22	Index: ‘Clinical Psychology Service’
Consultant/doctor (phone call)	£49	22	Index: ‘Follow-up, Non Face to Face’
Specialist nurse (phone call)	£70	22	Index: ‘Specialist Nursing, Asthma and Respiratory Nursing/Liaison, Adult, Non Face to Face’
Administrator/secretary (phone call)	£9	32	Assumed to be approximated by: nurse-led triage (cost per intervention)
**Scenario analysis: causal SAEs**			
Causal SAE #1	£3966	22	Weighted mean reference cost for ‘Ureteric or Bladder Disorders, with Interventions’
Causal SAEs #2 and #3	£1422	22	Weighted mean reference cost for ‘Non-Malignant Gastrointestinal Tract Disorders without Interventions’

Per-patient costs were calculated by multiplying each patient’s recorded HRU units by their corresponding price weight.

HRG, health resource group; HRU, health resource use; IMAG, diagnostic imaging; SAE, serious adverse event.

In light of an observed imbalance in hospital admissions deemed by the investigator to be unrelated to morphine, a post hoc costing scenario was undertaken in which hospitalisation costs were measured only by serious adverse events (SAEs) using industry-standard definitions considered to be related to morphine by site clinicians and study investigators, instead of the number of self-reported inpatient days which formed the base case a priori. All SAE reporting (including causality) was done while still blinded, except one SAE that was judged as having a causal relationship post hoc; this increased the number of causal SAEs (all in the morphine arm) from two to three, leading to a more conservative result than the fully blinded causality. In the scenario analysis, the hospital stay data were collected from the SAE reporting in the case report forms, which were verified from the clinical record by site investigators and study monitors, as opposed to inpatient stay data, which were sourced from patient questionnaires. Corresponding unit costs were sourced and approved for the three SAEs included in the scenario analysis by the Chief Investigators (reported in table 1).

All costs are reported in Great British Pounds for the financial year of 2021/2022, based on the most recent NHS reference costs available at the time of conducting the cost analysis.^22^

### Analysis

To account for missing data and the non-normal and skewed distribution of estimates, statistical tools from the validated *bootImpute* R programming package were used to estimate cost and QALY 95% CIs for means and differences in means between trial arms.^23 24^ These methods combined multiple imputation by chained equations, non-parametric bootstrapping and generalised linear model regression with a gamma distribution and a log link to account for estimate skewness.^23 25 26^

The regression model was controlled for age and sex, as well as study site and causal disease, and was consistent with the main clinical analysis.^8^ QALYs were additionally adjusted for baseline HSUV, as recommended by Manca *et al*.^18^

Estimates of the regression analyses from the simulated bootstrapped datasets and estimates from the pooled regression model were used to complete the cost-effectiveness analysis. As per NICE guidelines, the primary cost-effectiveness metric was the incremental cost-effectiveness ratio (ICER) measured in incremental costs per QALY gained (intervention vs control). The ICER was calculated alongside measures of parameter uncertainty in the form of a cost-effectiveness plane (CEP) scatterplot, a cost-effectiveness acceptability curve (CEAC), and a supplementary value of information (VoI) analysis containing an expected value of perfect information (EVPI) plot.^15^

### Concomitant medications

In contrast to the other patient-reported variables, concomitant medication data were collected from a CRF. Its analysis proved challenging for the following reasons.

- The concomitant medications for CRF had a large number of entries (n=1852).
- Drug names were recorded as a free-text variable, prone to erroneous and inconsistent spelling.
- Many values were impossible to interpret (eg, dose frequency was recorded as ‘Other’).
- Each entry needed to be cross-referenced with a unit cost from the BNF.
- Many concomitant medications were unrelated to the MABEL trial (eg, unrelated to breathlessness or morphine-related side effects).

To resolve these issues, the following steps were taken: the 1852 concomitant medication data entries were cleaned, from which 424 unique categorical drug values were extracted (including a missing/error term); then, the 424 drug names were classified into drug categories by a clinician and health economics lead on the MABEL trial team, 328 of which were classified as ‘not relevant’ (ie, unrelated to breathlessness or morphine-related side effects as judged by the clinical opinion of the trial investigators) and were therefore excluded from cost calculation to protect from imbalance introduced by low frequency but expensive unrelated prescription. Then, the unit costs of the remaining 96 drugs were identified by a web-scraping algorithm written in R using the *rvest* package with access to publicly available BNF data.^27^ It should be noted that the BNF website accepted web-scraping requests at the time of conducting the analysis (summer and autumn of 2024), but has since (as of August 2025) changed its end-user licensing agreement and no longer accepts such requests. Once the cost data from BNF was compiled, the observed medication cost was calculated for each non-excluded entry (n=444 out of n=1852) by multiplying the unit cost by the units used (as recorded in the CRF). At this point, there was still too much missing data to sum up medication costs by the patient, so the remaining data were imputed and bootstrapped using *bootImpute* in parallel to the patient-level data. This allowed imputed total per-patient concomitant medication costs to be calculated separately for each iteration of the simulated dataset.

### Patient and public engagement

No additional patient and public involvement was undertaken specifically for the trial’s health economic evaluation, beyond that of the main trial (see protocol for details).^8^

## Results

### Study population

140 participants formed the MABEL intention-to-treat population (see table 2). The Consolidated Standards of Reporting Trials flow diagram (reporting checklist) is provided in online supplemental figure 1. The protocol describes further details of the screening and randomisation processes.^8^

**Table 2:** Baseline characteristics—median (IQR), n (%) or mean±SD

Demographics and baseline variables	Placebo (n=67)	Morphine (n=73)	Total (n=140)
Age in years	70.3 (8.4)	70.6 (10.2)	70.5 (9.4)
Sex			
Male	45 (67%)	48 (66%)	93 (66%)
Female	22 (33%)	25 (34%)	47 (34%)
Ethnicity or origin			
White	63 (94%)	69 (95%)	132 (94%)
Asian/Asian British	2 (3%)	2 (3%)	4 (3%)
Black/African/Caribbean/Black British	1 (1%)	2 (3%)	3 (2%)
Other ethnic origin	1 (1%)	0	1 (<1%)
Primary diagnosis			
Chronic obstructive pulmonary disease	37 (55%)	40 (55%)	77 (55%)
Other non-malignant lung disease	30 (45%)	30 (41%)	60 (43%)
Heart disease	0	3 (4%)	3 (2%)
Cancer	0	1 (1%)	1 (<1%)
Body mass index (kg/m^2^)	27.4 (5.1)	27.1 (6.4)	27.2 (5.8)
Estimated glomerular filtration rate (mL/min/1.73 m^2^)	69.9 (19.1)	70.5 (16.7)	70.2 (17.8)
Resting BP (mm Hg)			
Systolic BP (mm Hg)	131.3 (23.3)	132.2 (20.5)	131.8 (21.8)
Diastolic (mm Hg)	75.1 (12)	74.8 (10.4)	75.0 (11.1)
Resting pulse (beats per minute)	79.4 (14.6)	80.3 (13)	79.9 (13.8)
Resting respiration rate (breaths/min)	20.0 (4.2)	20.6 (3.9)	20.3 (4)
Pulse oximetry	94.3 (2.6)	93.8 (3.4)	94.1 (3)
Comorbidities			
0	1 (1%)	3 (4%)	4 (3%)
1	15 (22%)	15 (21%)	30 (21%)
At least 2	51 (76%)	55 (75%)	106 (76%)
mMRC breathlessness scale (self-report)			
mMRC 1	1 (2%)	0	1 (<1%)
mMRC 2	1 (2%)	4 (6%)	5 (4%)
mMRC 3	43 (64%)	50 (69%)	93 (66%)
mMRC 4	22 (33%)	19 (26%)	41 (29%)
Australian Modified Karnofsky Performance Scale			
50—Considerable assistance, frequent medical care	5 (8%)	4 (6%)	9 (6%)
60—Occasional assistance, can care for most needs	26 (39%)	29 (40%)	55 (39%)
70—Cares for self; cannot carry on normal activity or work	25 (37%)	27 (37%)	52 (37%)
80—Normal activity with effort; some signs or symptoms	8 (12%)	11 (15%)	19 (14%)
90—Normal activity; minor signs or symptoms	2 (3%)	1 (1%)	3 (2%)
100—Normal; no complaints; no evidence of disease	1 (2%)	1 (1%)	2 (1%)
NRS			
NRS worst breathlessness	6.8 (1.9)	6.8 (1.5)	6.8 (1.7)
NRS distress due to breathlessness	5.1 (2.8)	5.3 (2.6)	5.2 (2.7)
NRS most severe cough	3.9 (2.7)	4.5 (2.9)	4.2 (2.8)
NRS pain	2.7 (2.8)	3.0 (2.8)	2.8 (2.8)
Short-Form 12			
Physical component	31.2 (6.3)	31.4 (6.5)	31.3 (6.4)
Mental component	44.4 (10.8)	49.3 (10.1)	46.9 (10.7)
ICEpop CAPability	0.67±0.12	0.64±0.14	0.65±0.13
EuroQol-five dimension-five level	0.56 (0.23)	0.60 (0.19)	0.58 (0.21)
EuroQol-Visual Analogue Scale	48 (21)	55 (16)	52 (19)
Epworth Sleep Scale	7.4 (5.4)	6.5 (4.9)	7.0 (5.2)
Karolinska Sleepiness Scale	7.4 (5.4)	6.5 (4.9)	7.0 (5.2)
Saint Louis University Mental Status Questionnaire	25.1 (3.2)	24.9 (4.1)	25.0 (3.7)
Average daily step count	7189 (3454)	6824 (2562)	6998 (3014)
Total time in moderate or vigorous activity—min/day	65 (56)	54 (39)	59 (48)

* Actigraphy monitor worn on non-dominant wrist.

BP, blood pressure; mMRC, modified Medical Research Council; NRS, numerical rating scale.

**Figure 1:**
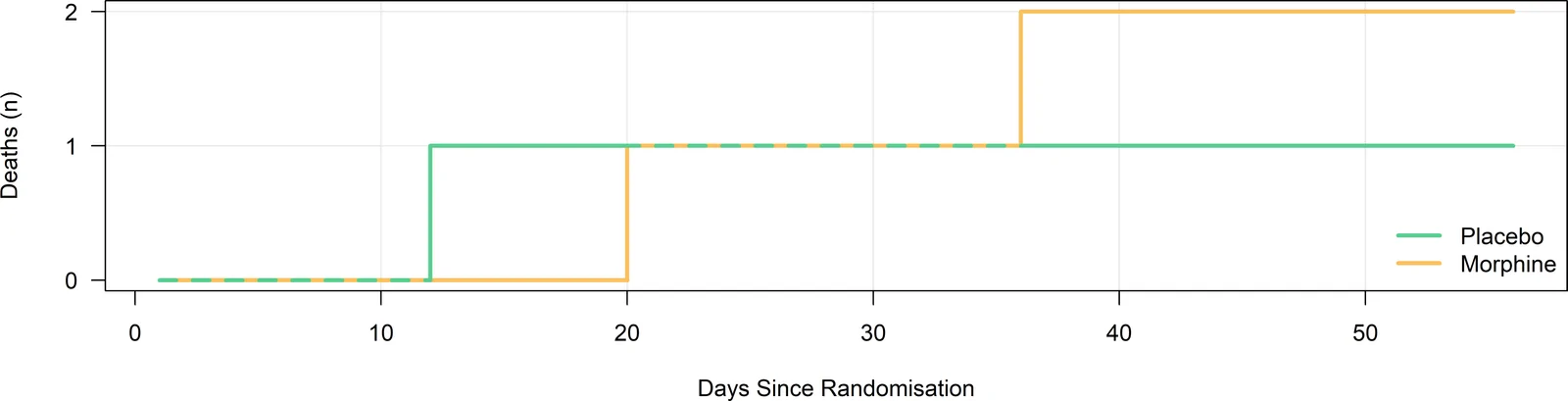
Mortality over time by study group.

Deaths over time by study group are presented in figure 1 and, in conjunction with EQ-5D-5L data, were used to calculate QALYs.

### Data quality

The percentage of patients in each arm with missing values is categorised by each patient-reported health economic variable and presented in online supplemental table 2. In these variables, missingness was below 20%. Unsurprisingly, missingness was highest for variables collected on day 56 since randomisation, followed by those gathered on day 28 and 0% for baseline measures. For both health outcomes and resources/costs, missingness is consistently slightly higher in the morphine arm (by up to 9 percentage points).

Concomitant medication data are summarised separately in online supplemental table 3. The table shows the number of entries in the CRF and the number and percentage of receiving patients by concomitant medication category.

### Health resource use

The sums of patient-reported health resources used by each study group, which were used to calculate hospital and community costs, are shown in table 3.

**Table 3:** Health resource use by study group—N (event count by study group) and mean±SD (per patient)

Variable	Day 28 placebo (n=67)		Morphine (n=73)		Day 56 placebo (n=67)		Morphine (n=73)	
	N	Mean±SD	N	Mean±SD	N	Mean±SD	N	Mean±SD
Inpatient and emergency								
Hospital inpatient stay (nights)	5	0.1±0.5	36	0.6±2.3	17	0.3±1.5	54	0.9±3.6
Hospice inpatient stay (nights)	7	0.1±0.9	0	0	7	0.1±0.9	0	0
Emergency assessments	2	0.0±0.2	1	0.0±0.1	2	0.0±0.2	3	0.0±0.2
Outpatient attendances								
Hospital consultant/doctor	10	0.2±0.4	4	0.1±0.2	18	0.3±0.6	13	0.2±0.5
Specialist nurse	20	0.3±1.2	6	0.1±0.3	30	0.5±1.5	6	0.1±0.3
Physiotherapist	17	0.3±1.2	11	0.2±1.0	18	0.3±1.3	12	0.2±1.1
Dietician	0	0	0	0	0	0	0	0
Occupational therapy	2	0.0±0.3	1	0.0±0.1	2	0.0±0.3	1	0.0±0.1
Social worker	0	0	0	0	0	0	0	0
Other outpatient	6	0.1±0.3	5	0.1±0.3	22	0.4±1.0	9	0.1±0.4
Hospice doctor	0	0	0	0	0	0	0	0
Hospice nurse	0	0	0	0	0	0	0	0
Day hospice	2	0.0±0.3	0	0	3	0.1±0.4	0	0
Other professional	0	0	0	0	0	0	0	0
Phone calls								
Hospital consultant/doctor	3	0.0±0.3	7	0.1±0.4	3	0.1±0.2	15	0.2±0.6
Specialist nurse	23	0.4±1.7	4	0.1±0.2	41	0.7±2.8	17	0.3±0.6
Administrator/secretary	1	0.0±0.1	1	0.0±0.1	6	0.1±0.4	4	0.1±0.3
Hospice consultant/doctor	0	0	0	0	0	0	0	0
Hospice specialist nurse	0	0	1	0.0±0.1	0	0	1	0.0±0.1
Hospice administrator/secretary	0	0	0	0	0	0	0	0

SD, standard deviation.

A disparity in resource use patterns is shown in table 3; patients in the morphine arm spent more inpatient nights than placebo (54 nights vs 17 nights by day 56), while the placebo arm used more outpatient and community services. While this could be explained by random variation, it is also possible that, due to small numbers, an imbalance in unrelated non-intervention-related SAEs has led to a spuriously high admission rate in the intervention arm. Therefore, we conducted a post hoc scenario analysis, where the self-reported inpatient stay nights were replaced with SAE hospitalisations recorded in the CRFs that were likely related to the intervention; three such SAEs were identified among three patients in the morphine arm (one due to urinary retention and two due to constipation) and 0/3 SAEs in the placebo arm (ie, none of the placebo arm SAEs were classed as causal).

### Regression analysis results

The summary statistics of the main MABEL economic variables are reported in table 4. The placebo and morphine arms present mean per-patient values and 95% CIs estimated via non-parametric bootstrapping.

**Table 4:** Summary statistics—mean estimate (95% confidence interval)

Variable	Placebo (n=67)	Morphine (n=73)	Difference	P value
Health outcomes				
EQ-5D-5L health utility (baseline)	0.48 (0.42 to 0.53)	0.51 (0.46 to 0.56)	–	–
EQ-5D-5L health utility (day 28)	0.49 (0.41 to 0.56)	0.55 (0.47 to 0.62)	0.05 (−0.02 to 0.13)	0.16
EQ-5D-5L health utility (day 56)	0.53 (0.47 to 0.58)	0.55 (0.46 to 0.62)	0.01 (−0.06 to 0.08)	0.74
ICECAP (baseline)	0.67 (0.64 to 0.70)	0.64 (0.61 to 0.67)	–	–
ICECAP (day 28)	0.67 (0.63 to 0.71)	0.63 (0.59 to 0.68)	−0.03 (−0.07 to 0.00)	0.08
ICECAP (day 56)	0.66 (0.61 to 0.70)	0.63 (0.56 to 0.67)	−0.04 (−0.08 to 0.01)	0.12
SF-6D (SF-12) (baseline)	0.58 (0.56 to 0.61)	0.63 (0.6 to 0.65)	–	–
SF-6D (SF-12) (day 28)	0.62 (0.59 to 0.66)	0.64 (0.60 to 0.69)	0.02 (−0.02 to 0.06)	0.28
SF-6D (SF-12) (day 56)	0.61 (0.58 to 0.64)	0.63 (0.59 to 0.68)	0.02 (−0.02 to 0.06)	0.30
QALYs				
QALYs (28 days)	0.055 (0.047 to 0.063)	0.062 (0.053 to 0.069)	0.005 (−0.003 to 0.013)	0.21
QALYs (56 days)	0.075 (0.065 to 0.084)	0.081 (0.071 to 0.090)	0.002 (−0.004 to 0.008)	0.51
Intervention costs (28 days)				
Morphine costs	£0	£2 (£2 to £2)	£2 (£2 to £2)	0.00
Laxative costs	£0	£4 (£4 to £4)	£4 (£4 to £4)	0.00
Hospital costs (28 days)				
Inpatient stay (nights)	£84 (£0 to £336)	£283 (£49 to £786)	£148 (−£32 to £359)	0.11
Emergency assessment (attendances)	£11 (£0 to £33)	£7 (£0 to £32)	−£1 (−£15 to £12)	0.85
Hospital consultant/doctor (attendances)	£45 (£22 to £70)	£23 (£6 to £48)	−£17 (−£39 to £5)	0.14
Specialist nurse (attendances)	£40 (£15 to £103)	£19 (£5 to £65)	−£19 (−£46 to £9)	0.18
Physiotherapist (attendances)	£38 (£5 to £101)	£28 (£1 to £95)	−£9 (−£40 to £24)	0.59
Dietician (attendances)	£0	£0	£0	0.99
Occupational therapy (attendances)	£4 (£0 to £18)	£3 (£0 to £15)	−£1 (−£7 to £6)	0.84
Social worker (attendances)	£0	£0	£0	0.99
Other outpatient (attendances)	£15 (£6 to £28)	£11 (£3 to £26)	−£2 (−£15 to £10)	0.71
Hospital consultant/doctor (phone calls)	£5 (£0 to £12)	£8 (£2 to £18)	£3 (−£2 to £9)	0.25
Specialist nurse (phone calls)	£31 (£5 to £92)	£11 (£2 to £59)	−£11 (−£33 to £11)	0.32
Administrator/secretary (phone calls)	£0 (£0 to £1)	£0 (£0 to £1)	£0 (£0 to £1)	0.83
Community costs (28 days)				
Hospice inpatient stay (nights)	£31 (£0 to £167)	£0 (£0 to £88)	−£22 (−£61 to £19)	0.28
Hospice doctor (attendances)	£0	£0	£0	0.99
Hospice nurse (attendances)	£0	£0	£0	0.99
Day hospice (attendances)	£3 (£0 to £13)	£0 (£0 to £11)	−£2 (−£6 to £2)	0.30
Other professional (attendances)	£0	£0	£0	0.95
Hospice consultant/doctor (phone calls)	£0	£0	£0	0.99
Hospice specialist nurse (phone calls)	£0 (£0 to £2)	£1 (£0 to £6)	£1 (−£2 to £4)	0.38
Hospice administrator/secretary (phone calls)	£0	£0	£0	0.99
Intervention costs (56 days)				
Morphine costs	£0	£4 (£3 to £4)	£3 (£3 to £4)	0.00
Laxative costs	£0	£7 (£7 to £8)	£7 (£7 to £8)	0.00
Hospital costs (56 days)				
Inpatient stay (nights)	£190 (£13 to £790)	£431 (£75 to £1471)	£260 (−£131 to £733)	0.21
Emergency assessment (attendances)	£17 (£0 to £48)	£23 (£0 to £58)	£6 (−£13 to £24)	0.55
Hospital consultant/doctor (attendances)	£87 (£47 to £153)	£73 (£31 to £140)	−£10 (−£44 to £26)	0.58
Specialist nurse (attendances)	£67 (£26 to £160)	£31 (£7 to £122)	−£28 (−£61 to £6)	0.10
Physiotherapist (attendances)	£44 (£9 to £128)	£37 (£3 to £144)	−£9 (−£45 to £30)	0.65
Dietician (attendances)	£0	£0	£0	0.98
Occupational therapy (attendances)	£4 (£0 to £12)	£2 (£0 to £7)	−£1 (−£8 to £7)	0.85
Social worker (attendances)	£0	£0	£0	0.98
Other outpatient (attendances)	£46 (£19 to £93)	£29 (£10 to £79)	−£14 (−£39 to £12)	0.30
Hospital consultant/doctor (phone calls)	£7 (£1 to £19)	£16 (£7 to £34)	£9 (£1 to £17)	0.03
Specialist nurse (phone calls)	£61 (£17 to £193)	£40 (£14 to £143)	−£9 (−£42 to £25)	0.58
Administrator/secretary (phone calls)	£2 (£0 to £4)	£1 (£0 to £4)	£0 (−£2 to £1)	0.80
Community costs (56 days)				
Hospice inpatient stay (nights)	£35 (£0 to £126)	£0	−£27 (−£78 to £26)	0.30
Hospice doctor (attendances)	£0	£0	£0	0.98
Hospice nurse (attendances)	£0	£0	£0	0.98
Day hospice (attendances)	£4 (£0 to £14)	£0	−£5 (−£16 to £5)	0.30
Other professional (attendances)	£0	£0	£0	0.98
Hospice consultant/doctor (phone calls)	£0	£0	£0	0.98
Hospice specialist nurse (phone calls)	£0	£1 (£0 to £4)	£1 (−£1 to £4)	0.35
Hospice administrator/secretary (phone calls)	£0	£0	£0	0.98
Concomitant medications				
Concomitant medications (28 days)	£20 (£0 to £127)	£18 (£1 to £116)	−£5 (−£64 to £58)	0.88
Concomitant medications (56 days)	£65 (£10 to £338)	£44 (£8 to £309)	−£19 (−£84 to £49)	0.57
Causal SAEs (scenario analysis)				
Causal SAE costs (28 days)	£0	£0	£0	–
Causal SAE costs (56 days)	£0	£82 (£0 to £234)	£73 (−£14 to £169)	0.10
Total costs				
Total costs (28 days, base case)	£350 (£133 to £669)	£464 (£151 to £1280)	£102 (−£216 to £517)	0.57
Total costs (28 days, scenario)	£256 (£113 to £486)	£144 (£52 to £405)	−£58 (−£154 to £50)	0.28
Total costs (56 days, base case)	£520 (£286 to £833)	£572 (£243 to £1082)	£24 (−£395 to £552)	0.92
Total costs (56 days, scenario)	£384 (£234 to £660)	£261 (£144 to £535)	−£48 (−£173 to £98)	0.50

The difference column presents regression estimates and 95% confidence intervals of the mean effects of the investigational medicinal product on each variable, adjusted for age, sex, centre and disease type. QALYs are additionally adjusted for baseline utility.

EQ-5D-5L, EuroQol 5-dimension 5-level score; ICECAP, ICEpop CAPability measure; QALYs, quality-adjusted life years; SAE, serious adverse event; SF-6D (SF-12), 6-dimension health utility index derived from the 12-Item Short-Form Health Survey.

Overall, table 4 presents no statistically significant differences in health outcome measures or costs between trial arms, with only two exceptions:

1. IMP and NIMP costs, which are set to £0 in the placebo arm but are £4 (95% CI £3 to £4) and £7 (95% CI £7 to £8) in the morphine arm, respectively.
2. Hospital consultant/doctor phone calls have a small but statistically significant (p=0.03) difference, being £9 (95% CI £1 to £17) higher in the morphine arm relative to placebo.

### Cost-effectiveness analysis results

The 56-day ICER for the MABEL intervention was estimated as a function of the adjusted difference in QALYs and total costs, resulting in $\frac{\Delta Costs}{\Delta QALYs}=\frac{£24\;}{0.002}=£12,000$ per QALY gained in the base case. In the scenario analysis, morphine lowers costs by −£48 (95% CI −£173 to £98); therefore, the ICER cannot be calculated, and the intervention dominates standard care.

**Figure 2:**
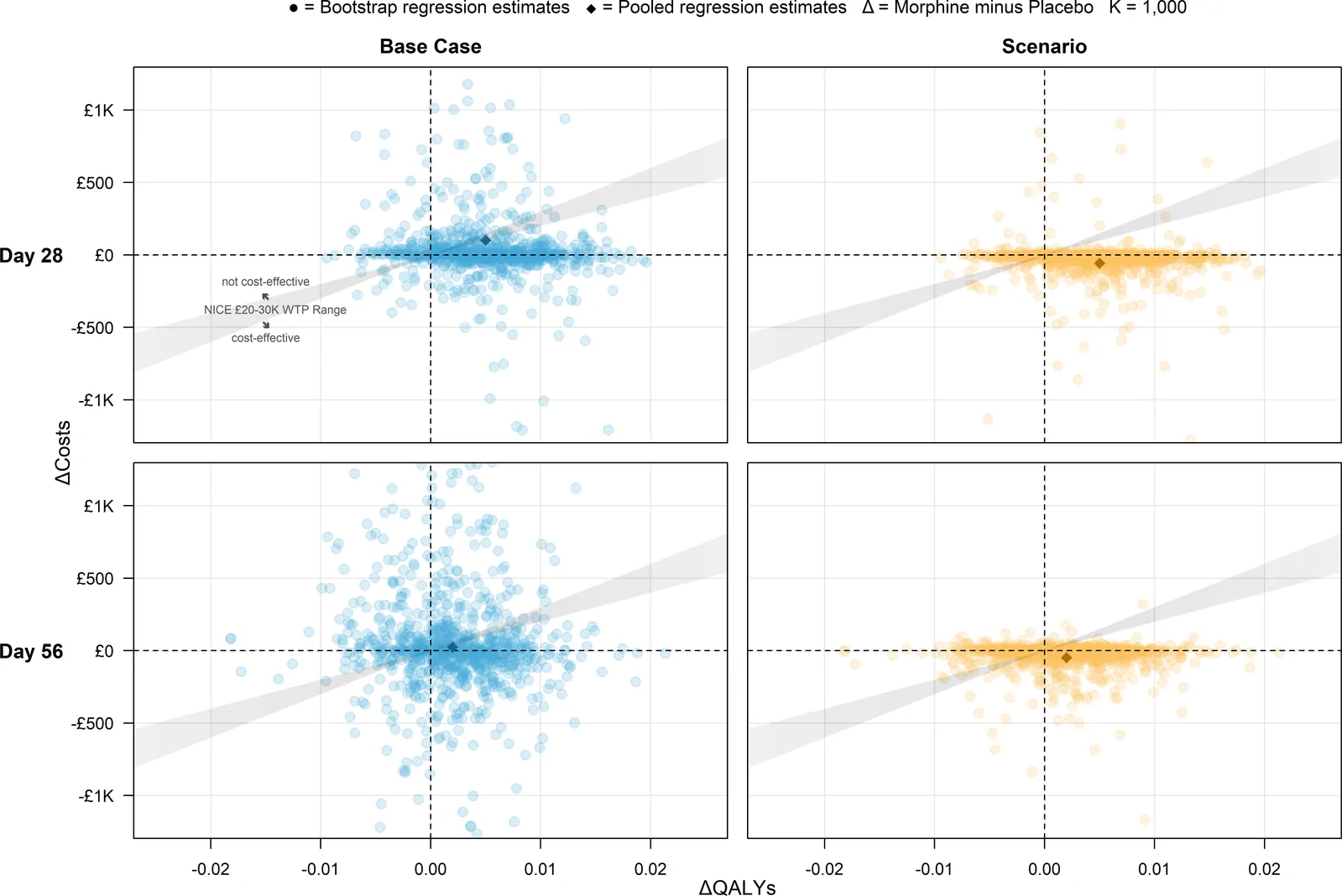
Cost-effectiveness planes.

Uncertainty around the ICER results is presented in the CEPs in figure 2, which plots the adjusted regression estimates for morphine’s effect on QALYs and total costs, separated by day 28 versus day 56 and base case versus scenario (non-causal SAEs removed from costing). For comparison and readability of results by time point and scenario, each CEP in figure 2 displays the same scale and grid, resulting in the omission of many bootstraps with large estimated cost differences between trial arms. The full ranges of bootstrapped estimates are presented in online supplemental figure 2, with estimated cost differences plotted on a log scale. Of note are highly positively skewed cost difference outliers present in the day 28 bootstraps, which are most likely errors in the imputation or cost regression for those particular bootstraps. No such outliers were observed in any of the QALY results or the day 56 cost difference estimates; presumably, this is due to more complete data, less prone to imputation or regression algorithm errors. The distributions of bootstrapped differences in costs and QALYs are summarised in table 5 as percentages of simulated results in each quadrant of the corresponding CEP.

**Table 5:** Summary of cost-effectiveness results

Outcome	Base case	Scenario	
56-day probability morphine leads to:			CEP quadrant
Higher costs, better health	34%	20%	Northeast
Higher costs, worse health	18%	12%	Northwest
Lower costs, better health	34%	48%	Southeast
Lower costs, worse health	14%	20%	Southwest
Probability morphine is cost-effective:			Willingness to pay threshold
By day 28	66%	87%	£20 000
	69%	86%	£30 000
By day 56	54%	73%	£20 000
	55%	72%	£30 000

CEP, cost-effectiveness plane.

**Figure 3:**
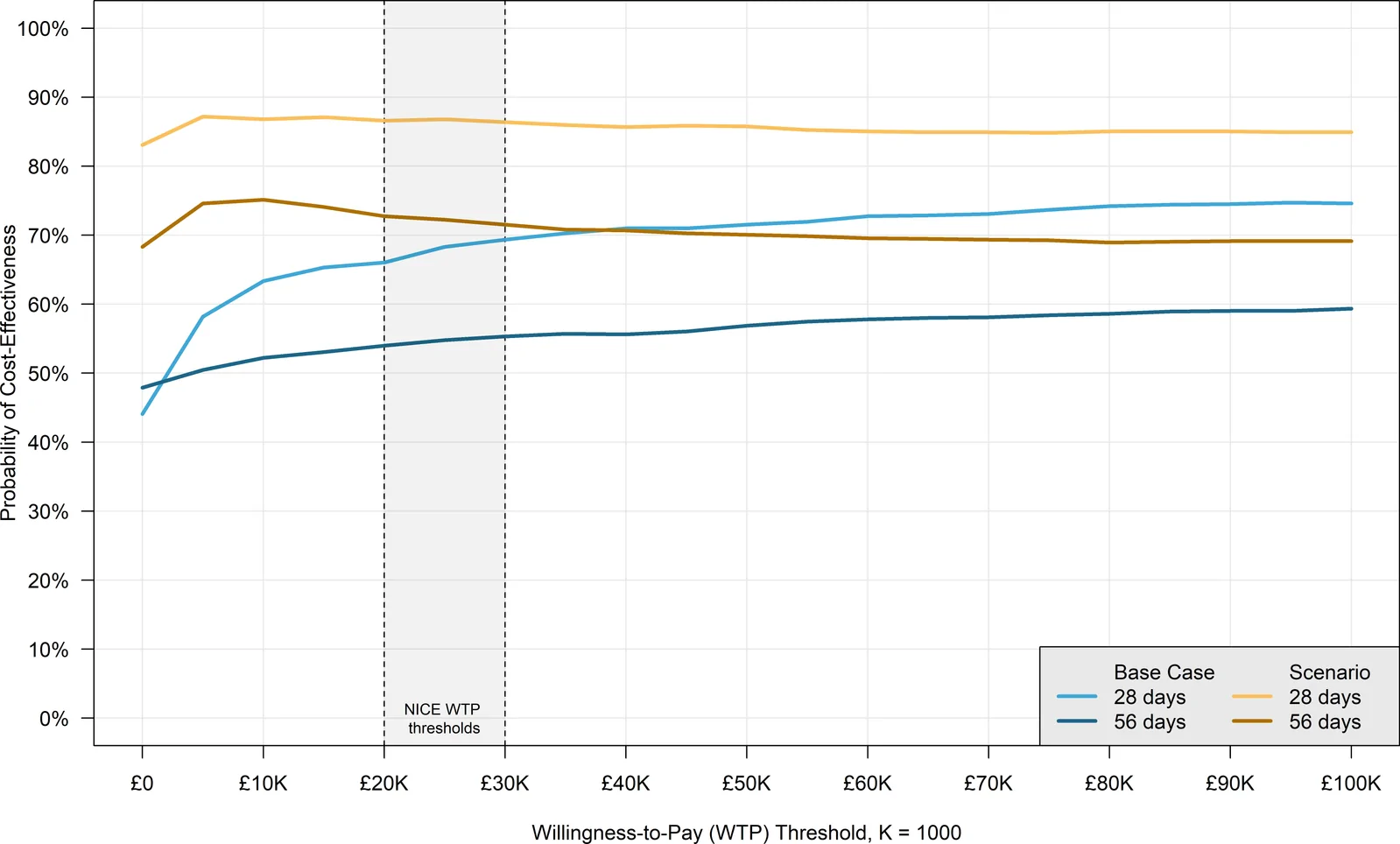
Probability of cost-effectiveness.

The CEAC, which plots the probability of morphine’s cost-effectiveness compared with standard care against a WTP threshold stratified by time point and scenario, is presented in figure 3. Selected CEAC points (probabilities of cost-effectiveness using NICE WTP thresholds of £20 000 and £30 000) are also presented in table 5. Due to the CEAC being a percentage of cost-effective bootstraps at a given WTP, it was not judged to be sensitive to the cost outliers present in the day 28 bootstraps, which were consequently not removed from CEAC calculations.

A VoI analysis was conducted alongside the CEAC calculations. The VoI results are presented in online supplemental figure 3, which shows the EVPI plotted against the WTP threshold, stratified by time point and scenario. Since the VOI plots the mean EVPI at each WTP, it was sensitive to the day 28 cost outliers. As a result, the EVPI was calculated from a truncated distribution of the middle 95% of incremental net benefit values, yielding results far more consistent with the CEACs in figure 3 and regression results in table 4; for consistency, this was applied to all four EVPI curves.

## Discussion

The analysis suggests that long-acting morphine may be cost-effective for chronic breathlessness, at a probability consistent with the previous health economic evaluation in the context of COPD.^7^ In the scenario-based post hoc analysis, the probability of cost-effectiveness becomes greater. However, the adjusted results of MABEL’s health economic analysis show no overall statistically significant difference in costs or health outcomes between the placebo and long-acting arms, and the cost-effectiveness estimates are highly uncertain. This is consistent with its main clinical results, which show that long-acting low-dose oral morphine had no statistically significant effect on patient-reported worst breathlessness in people with chronic breathlessness.^9^ The ICER is difficult to interpret when taken in isolation due to the small sample size and effect of a likely random imbalance in deaths; therefore, we suggest that interpretation should focus on the cost-consequence analysis, as this allows us to unpick possible relationships between adverse events, morphine, costs and patterns of health service use. Furthermore, day 28 cost difference estimates should be taken with caution due to possible errors in imputation or regression producing a small number of highly skewed outliers.

Despite the lack of statistical significance in the cost analysis, an interesting divergence in patient-reported HRU patterns was observed between trial arms. More outpatient services were used in the placebo arm, while the morphine arm patients used more inpatient services. These inpatient admissions appeared to be imbalanced and unexplained by adverse events plausibly related to morphine. The scenario analysis proved a useful tool to rebalance this in case the trial’s sample size exacerbated the imbalance. However, a limitation of the scenario analysis stems from it being conducted post hoc and judging one of the SAEs as causal only after unblinding, unlike the fully double-blinded base case analysis. However, observational studies of cohorts of people with advanced COPD and interstitial lung diseases (conditions that 55% and ~40% of the MABEL trial population had, respectively) demonstrated no excess hospital admissions or deaths in people taking opioids at less than 30 mg morphine-equivalent doses per day.^28 29^ This is despite having very severe respiratory disease; all participants in these cohorts are on long-term oxygen therapy. Of note in the MABEL trial, where hospital admission was attributed to morphine, two of the three admissions were related to constipation. This emphasises the importance of close attention to bowel care in anyone taking morphine—this is a potentially avoidable situation, and in retrospect, the blinded laxative in the MABEL trial should have included a stimulant as well as a softener. Furthermore, we need to consider whether some infectious adverse events might be related to morphine. There is a theoretical concern about the impact of opioids on the immune system, although the effect on clinical outcomes is unknown. However, we found no evidence that participants in the morphine arm experienced more infectious events than the placebo arm, making it unlikely that relatedness was misclassified in this regard.

The MABEL analysis of health outcomes produced some unexpected results. Adjusted point estimates for morphine’s impact on EQ-5D and SF-12 scores on day 56 are positive, while those for ICECAP are negative. While not statistically significant, this is still the opposite trend to what we expected before data analysis, as it suggests that participants taking morphine are *less* capable. However, this result could be consistent with the HRU analysis, wherein morphine arm participants used more inpatient services (eg, hospitalisation) and fewer outpatient services relative to placebo. Hospital inpatient stays would be expected to impair capability, although this is not connected to and does not suggest morphine-related cognitive bluntness. The conflict with EQ-5D-5L and SF-12 scores might stem from the pain reduction morphine provides, but the clinically and statistically significant improvement in pain in the morphine arm at day 7 (mean difference −0.92, 95% CI −1.70 to –0.13, p=0.2) was not sustained throughout the 56 days of the MABEL trial, other than a small and insignificant improvement.^9^ Undoubtedly, the contrasts between these health outcomes highlight the importance of future research to find the most appropriate measures of health outcomes in the context of advanced illness. It is also interesting to note the cost implications of outpatient and community care. People with moderate to severe breathlessness due to chronic medical conditions are high users of all aspects of healthcare (community, emergency and hospital), with costs also driven by community care health service needs.^6^

We highlight several strengths and weaknesses. While the robustness of patient-reported data collection was a significant strength of the analysis, the difficulties in analysing data from concomitant medications proved to be a non-trivial study limitation. We also acknowledge the limitations of our study design: not accounting for placebo side-effect impacts on concomitant medication and no management of these in this context. Nevertheless, MABEL’s analysis of concomitant medication was consistent with literature showing that such medication costs constitute a small percentage of total costs (and thus have a negligible impact on cost-effectiveness) and are characterised by high data missingness, with the potential to improve research quality and data collection efficiency.^30^ Future trials based in the UK could avoid these issues and improve research by ensuring drug names are not entered as free text and instead selected or searched from a pre-specified list corresponding to entries in the BNF, similar to the framework developed and recommended by the US-based ‘ASPirin in Reducing Events in the Elderly’ study.^31^ This would save researchers’ time, minimise errors created by spelling or data entry mistakes and ensure observed drugs can be matched with the price weights needed to estimate medication costs. Furthermore, an improved concomitant medication CRF design could ensure a more consistent way of recording the dosage to let researchers easily identify how many units of a given drug each patient has taken over the study time horizon. The challenge in this patient population (nearly all had at least one comorbidity) is that most are taking multiple medications.

As noted in the methods, a few items outlined in the HEAP were not implemented in the final analysis, including the societal-perspective analysis (eg, productivity loss due to illness) calculations and QALYs derived from non-EQ-5D-5L measures (eg, calculated from SF-6D instead) due to COVID-19-related delays and researcher availability. Furthermore, given the literature on COPD, a subgroup analysis specific to this disease was not included but would be useful in studies with larger sample sizes. These are limitations of this study, and including these items in future research would be recommended.

There are significant limitations in the ICER analysis as described above. There are also challenges when health economics are applied within a palliative context. It is likely that if morphine were a new drug developed for the management of breathlessness, a licence would not be granted based on efficacy, making cost-effectiveness irrelevant. However, in the postmarketing context of morphine being repurposed for use in chronic breathlessness, we estimate that if morphine were implemented for this indication, then, on balance, the use of morphine would improve net health gain from our current NHS budget. However, this does not necessarily mean it is the correct thing to do from a clinical point of view.

A VoI analysis was conducted alongside the CEAC calculations (see online supplemental figure 3), which resulted in a relatively low value of perfect information in the NICE £20–30K WTP range (£100–500 and <£50 of net monetary benefit in the base case and scenario analysis, respectively). The VoI implications are twofold: (1) MABEL is a robust trial, so further research in this area would likely point to similar decision recommendations, and (2) the consequence of making the wrong decision is low, which is consistent with the non-statistically significant clinical results. Day 28 EVPI curves should be interpreted with caution due to outliers present in the bootstraps (see online supplemental figure 2) despite the adjustment applied. It should also be noted that the VoI analysis does not reflect uncertainty in the framing of the decision problem (eg, choice of patient-relevant clinical outcome), which may be important for further research.

## Conclusion

While the MABEL health economic analysis must be taken with a degree of caution, as it does not show statistically significant effects of morphine on costs or health outcomes in people with chronic breathlessness, the scenario analysis shows a high probability of cost-effectiveness stemming from small non-statistically significant improvements in health outcomes and cost savings due to lower outpatient and community health resource usage. However, the ICER is difficult to interpret due to the small sample size and the effect of a likely random imbalance in deaths, and interpretation should focus on the cost-consequence analysis. Despite these limitations, this study presents high-quality clinical trial results, contributes to the health-economic literature on morphine and breathlessness and provides robust estimates that can be used in future economic modelling.

## Supplementary material

10.1136/bmjopen-2025-102124 online supplemental file 1

10.1136/bmjopen-2025-102124 online supplemental file 2

10.1136/bmjopen-2025-102124 online supplemental file 3

10.1136/bmjopen-2025-102124 online supplemental file 4

## Data Availability

Data are available upon reasonable request.
