# Supplementary material for: Morphine for chronic breathlessness (MABEL) in the UK: a health economic evaluation of a multisite, parallel-group, dose titration, double-blind, randomised, placebo-controlled trial
Source: BMJ Open. 2025 Nov 4;15(11):e102124. doi: 10.1136/bmjopen-2025-102124 (PMC12587952; doi:10.1136/bmjopen-2025-102124)
Supplement: online supplemental file 1 [file bmjopen-15-11-s001.pdf]

Docusign Envelope ID: 3C5C97E1-1267-4F58-8E04-778B86FC7992

MABEL

Health Economic Trial Analysis

Version No	1.2
Date Finalised	21.02.2024
Author(s)	Marek Atter, Professor Peter Hall
CI Name(s)	Professor Miriam Johnson, Professor Marie Fallon
CI Email address	*miriam.johnson@hyms.ac.uk*, *marie.fallon@ed.ac.uk*

Signatures	
Trial Lead Health Economist: Professor Peter Hall	Date: DocuSigned by: Peter Hall 8DC59C57977E447... 20-Dec-2024 | 09:23 CET
Chief Investigator: Professor Miriam Johnson	Date: Miriam Johnson box SIGN 4W6ZZQZ6-4LP5ZLL9 13 Jan 2025
Chief Investigator: Professor Marie Fallon	Date: Signed by: Marie Fallon ABF737D8F487439... 19-Dec-2024 | 14:58 GMT

Document Control		
Version No	Date	Summary of Revisions
1.0	1.12.2023	Initial Creation by author Marek Atter
1.1	23.12.2023	Additions by Peter Hall
1.2	21.02.2024	Response to comments by David Meads

Supporting Internal Documents	Version No
MABEL Protocol	1.3
MABEL Statistical Analysis Plan (SAP)	1.0

Docusign Envelope ID: 3C5C97E1-1267-4F58-8E04-778B86FC7992

1. Table of Contents

1. Table of Contents ... 2
2. Abbreviations ... 3
3. Introduction ... 4
3.1. Study Details ... 4
3.2. Background and Context ... 4
3.3. Health Economic Research Hypothesis, Questions and Objectives ... 4
4. Economic Principles ... 5
4.1. Time Horizon ... 5
4.2. Health Outcomes ... 5
4.3. Cost Outcomes ... 5
4.4. Cost-Effectiveness ... 5
5. Methods ... 6
5.1. Calculating QALYs ... 6
5.2. Calculating Costs ... 6
5.3. Currency and Conversion ... 7
5.4. Data Analysis ... 7
6. References ... 7

2. Abbreviations

Abbreviation	Full Name
BMI	Body Mass Index
CEAC	Cost Effectiveness Acceptability Curve
CEP	Cost-Effectiveness Plane
CI	Confidence Interval
CMM	Continuous Cardiac Monitoring
CPI	Consumer Price Index/Indices.
CRF	Case Report Form
CTIMP	Clinical Trials of Investigational Medicinal Products
DN	District Nurse
ECTU	Edinburgh Clinical Trials Unit
ED	Emergency Department
EQ-5D-3L	Euroqol Quality of Life Survey [3 Level version]
EQ-5D-5L	Euroqol Quality of Life Survey [5 Level version]
GLM	Generalised Linear Modelling
GP	General Practitioner
HE	Health Economics
HEAP	Health Economic Analysis Plan
HRU	Healthcare Resource Utilisation
HSUV	Health State Utility Value
ICER	Incremental Cost Effectiveness Ratio
IMP	Investigational Medicinal Product
INMB	Incremental Net Monetary Benefit
**MABEL**	**Morphine And BrEathLessness trial**
MICE	Multiple Imputation by Chained Equations
MRM	Modified Release Morphine
NHS	[The UK] National Health Service
NICE	[The] National Institute for [Health and] Care Excellence
NIHR	National Institute for Health Research
NRS	Numerical Rating Scale
OLS	Ordinary Least Squares
ONS	Office for National Statistics.
PN	Practice Nurse
POD	Post Operative Day
POD	Post Operative Day
PROM	Patient-Reported Outcome
PSA	Probabilistic Sensitivity Analysis
PSS	[The UK] Personal Social Services
QALY	Quality Adjusted Life Year
QoL	Quality of Life
RQ	Research Question
SAP	Statistical Analysis Plan
SOP	Standard Operating Procedure
UK	United Kingdom
VoI	Value of Information
WTP	Willingness-to-Pay [Threshold]

Docusign Envelope ID: 3C5C97E1-1267-4F58-8E04-778B86FC7992

3. Introduction

3.1. Study Details

MABEL is a "parallel group, double-blind, randomised, placebo-controlled trial comparing the effectiveness and cost effectiveness of low dose oral modified release morphine versus placebo on breathlessness in people with chronic breathlessness" [Protocol]. The economic component of MABEL shall include a cost-consequence analysis, which reports costs and consequences of each trial arm, and a cost-effectiveness analysis, which measures incremental costs against incremental health benefits associated with using the Investigational Medicinal Product (IMP).

3.2. Background and Context

The study background and context are provided by the MABEL Protocol:

> *"CHRONIC BREATHLESSNESS is frightening and devastating, prevalent in chronic progressive illnesses. It affects >90% of people with advanced lung cancer, chronic obstructive pulmonary disease (COPD), interstitial lung disease (ILD) and >80% with heart failure. Breathlessness is mostly triggered or worsened by exertion or anxiety. Despite treatment of underlying disease(s), chronic breathlessness often persists - Chronic Breathlessness Syndrome – and is neglected despite evidence that non-pharmacological interventions can help and pharmacological interventions have potential.* […] *THERE IS AN URGENT NEED to identify if* [modified release morphine (MRM)] *improves chronic breathlessness, and, if so, to identify the clinical care pathway that will become the new standard of care"*

3.3. Health Economic Research Hypothesis, Questions and Objectives

Health Economics (HE) Hypothesis: MRM for breathlessness in people with chronic breathlessness is a cost-effective intervention at the UK-specific Willingness-to-Pay (WTP) thresholds defined by the National Institute for Health and Care Excellence (NICE), which are £20,000 and £30,000 per Quality-Adjusted Life Year (QALY).[1]

HE Research Question: What are the differences and drivers of differences in costs and health outcomes between MRM and placebo?

HE Objectives:

1. *Primary:* To investigate the cost-consequence and cost-effectiveness of MRM for breathlessness in people with chronic breathlessness from a National Health Service (NHS) and Personal Social Services perspective, in accordance with NICE guidelines.[1]

2. *Secondary:* To investigate the cost-consequence and cost-effectiveness of MRM for breathlessness in people with chronic breathlessness from a societal perspective, which will

Docusign Envelope ID: 3C5C97E1-1267-4F58-8E04-778B86FC7992

include charitable costs, patient out-of-pocket expenses, lost productivity, welfare support and informal care, provided feasibility of data collection is confirmed in the internal pilot.

4. Economic Principles

4.1. Time Horizon

The Assessment and Activity Schedule in the protocol shows that all relevant health economic data will be collected at the following timepoints: Baseline (between Day -8 and Day 0), Day 28, and Day 56. The data will be sourced from the following questionnaires:

- EQ-5D-5L and EQ-VAS
- ICECAP-SCM
- SF-12
- Health Resource and Utilisation questionnaire

Therefore, the time horizon assumed by the health economic analysis shall be 56 days.

4.2. Health Outcomes

As per NICE guidelines, the health outcome of interest will be the QALY, which is an “index of survival that is adjusted to account for the patient's quality of life” best calculated using the EQ-5D measure.[1,2]

In light of the evolving discussion in the scientific literature about the interpretation of the QALY in end-of-life settings, the EQ-VAS, SF-6D (derived from SF-12) and the ICECAP-SCM will be used as alternative measures in the sensitivity analysis.[3,4]

4.3. Cost Outcomes

Following NICE guidelines, the base case cost analysis will use a UK NHS and Personal Social Services perspective, which includes the costs of secondary care, primary care and community NHS activity. As part of the sensitivity analysis, the costs will also be analysed through a societal perspective, which includes charitable costs, patient out-of-pocket expenses, lost productivity, welfare support and informal care, subject to feasibility of data collection.

4.4. Cost-Effectiveness

The main cost-effectiveness outcome reported will be the Incremental Cost-Effectiveness Ratio (ICER), which is defined as the ratio of the differences in mean costs and outcomes associated with the IMP.

Docusign Envelope ID: 3C5C97E1-1267-4F58-8E04-778B86FC7992

ICERs will be presented in terms of cost per QALY gained by the IMP. Uncertainty around the cost-effectiveness of the IMP will be presented graphically on a Cost-Effectiveness Plane (CEP) and a Cost Effectiveness Acceptability Curve (CEAC), following NICE guidelines.[1] As per NICE recommendations, the cost-effectiveness analysis will consider Willingness-to-Pay (WTP) ratios of £20,000 and £30,000 when plotting CEACs and judging whether or not the IMP is cost-effective from the NHS perspective.[1]

5. Methods

5.1. Calculating QALYs

QALYs will be calculated individually for each patient by using the following steps:

1. Calculating Health State Utility Values (HSUVs) from EQ-5D-5L Patient-Reported Outcome Measure (PROM) questionnaire data at each time point using a validated mapping function by Hernández Alava et al.[5]
2. Calculating 56-day QALYs as a function of HSUVs and their corresponding timepoints using the area-under-the-curve method outlined by Manca et al.[6]

Similarly to the EQ-5D-5L data, the alternative measures will be converted into tariff values and used to calculate QALYs using corresponding validated methods. The SF-6D will be converted into HSUVs using validated ProCore software provided by QualityMetric, while the ICECAP-SCM measure will be converted into tariff values for its capability measure, which ranges from 0 (no capability) to 1 (full capability), using its published value set.[7,8]

QALYs will be adjusted for baseline HSUV, as outlined by Manca et al.[6]

5.2. Calculating Costs

All HRU items included in the analysis, which include HRU data from the questionnaire and in the trial Case Report Forms (CRFs) collected on or before Day 56, will be counted for each patient. Then, each item's count will be multiplied by a corresponding unit cost.

Unit costs for HRU items will be sourced from the most up-to-date NHS National Cost Collection at the time of analysis.[9] Where an HRU item is not found in the National Cost Collection, it will be sourced from the most recent Unit Costs of Health and Social Care Manual available.[10] If neither of these sources contain the unit cost of an HRU item, the cost will be obtained by either searched in published literature via PubMed or sourced by consulting a relevant clinical professional, depending on which option is more suitable or feasible for a given HRU item.

Docusign Envelope ID: 3C5C97E1-1267-4F58-8E04-778B86FC7992

5.3. Currency and Conversion

All costs in this analysis will be reported in GBP (£) with 2023 as the base year. Pre-2023 unit costs will be converted into 2023 GBP using the health-specific consumer price index (CPI) reported in the Office for National Statistics' consumer price inflation tables.[11]

5.4. Data Analysis

Means and standard deviations of the main outcomes (HRU, costs, and QALYs), along with mean differences between trial arms, will be calculated using the R statistical programming software.[12]

Costs will be reported separately for each HRU category and summed together as total costs. Initial cost assignment will be undertaken blind to randomisation. Median, inter-quartile range and range of healthcare utilisation count data and costs will also be reported where relevant.

The effect of the IMP on costs and QALYs will be estimated using an appropriate regression model chosen upon data inspection. The choice of model will be guided by the algorithm recommended by Manning and Mullahy.[13] The regression will adjust for baseline characteristics (age and sex), as well as the following terms consistent with the analysis outlined in the statistical analysis plan (SAP):

- Baseline Numerical Rating Scale (NRS) worst breathlessness
- Site
- Causal disease

95% Confidence Intervals (CIs) will be calculated using non-parametric bootstrapping to account for the right-skewed distributions of cost variables.

Missing data handling will involve imputing missing values using an imputation method determined at time of analysis, which is consistent with the SAP.

In line with the SAP, sensitivity analysis will include a complier average causal effect (CACE) analysis to account for non-compliance not considered in the primary intention-to-treat analysis.[14] Treatment adherence is defined in the SAP as a binary variable with a clinically relevant adherence of ≥70%.

6. References

1. National Institute for Health and Care Excellence. *Guide to the Methods of Technology Appraisal 2013*. National Institute for Health and Care Excellence (NICE); 2013. Accessed May 22, 2023. http://www.ncbi.nlm.nih.gov/books/NBK395867/

2. National Institute for Health and Care Excellence. Position statement on use of the EQ-5D-5L value set for England. NICE. Accessed January 3, 2024. https://www.nice.org.uk/about/what-we-do/our-programmes/nice-guidance/technology-appraisal-guidance/eq-5d-5l

Docusign Envelope ID: 3C5C97E1-1267-4F58-8E04-778B86FC7992

3. Brazier J, Roberts J, Deverill M. The estimation of a preference-based measure of health from the SF-36. *J Health Econ*. 2002;21(2):271-292. doi:10.1016/S0167-6296(01)00130-8

4. Canaway A, Al-Janabi H, Kinghorn P, Bailey C, Coast J. Development of a measure (ICECAP-Close Person Measure) through qualitative methods to capture the benefits of end-of-life care to those close to the dying for use in economic evaluation. *Palliat Med*. 2017;31(1):53-62. doi:10.1177/0269216316650616

5. Hernández Alava M, Pudney S, Wailoo A. Estimating the Relationship Between EQ-5D-5L and EQ-5D-3L: Results from a UK Population Study. *PharmacoEconomics*. 2023;41(2):199-207. doi:10.1007/s40273-022-01218-7

6. Manca A, Hawkins N, Sculpher MJ. Estimating mean QALYs in trial-based cost-effectiveness analysis: the importance of controlling for baseline utility. *Health Econ*. 2005;14(5):487-496. doi:10.1002/hec.944

7. Huynh E, Coast J, Rose J, Kinghorn P, Flynn T. Values for the ICECAP-Supportive Care Measure (ICECAP-SCM) for use in economic evaluation at end of life. *Soc Sci Med*. 2017;189:114-128. doi:10.1016/j.socscimed.2017.07.012

8. QualityMetric. Health Surveys. We Measure Health. Accessed January 24, 2024. https://www.qualitymetric.com/health-surveys/

9. National Health Service (UK). National Cost Collection: National schedule of NHS costs - Year 2021/22 - NHS trusts and NHS foundation trusts. Accessed December 12, 2023. https://www.england.nhs.uk/wp-content/uploads/2023/04/2_National_schedule_of_NHS_costs_FY21-22_v3.xlsx

10. Jones KC, Weatherly H, Birch S, et al. *Unit Costs of Health and Social Care 2022 Manual*. Personal Social Services Research Unit (University of Kent) & Centre for Health Economics (University of York); 2023. doi:10.22024/UniKent/01.02.100519

11. Office for National Statistics (UK). UK Consumer Price Inflation Tables. Accessed March 17, 2023. https://www.ons.gov.uk/economy/inflationandpriceindices/datasets/consumerpriceinflation/current

12. The R Foundation. R: The R Project for Statistical Computing. Accessed January 4, 2024. https://www.r-project.org/

13. Manning WG, Mullahy J. Estimating log models: to transform or not to transform? *J Health Econ*. 2001;20(4):461-494. doi:10.1016/s0167-6296(01)00086-8

14. Dunn G, Maracy M, Tomenson B. Estimating treatment effects from randomized clinical trials with noncompliance and loss to follow-up: the role of instrumental variable methods. *Stat Methods Med Res*. 2005;14(4):369-395. doi:10.1191/0962280205sm403oa
