# Supplementary material for: Morphine for chronic breathlessness (MABEL) in the UK: a health economic evaluation of a multisite, parallel-group, dose titration, double-blind, randomised, placebo-controlled trial
Source: BMJ Open. 2025 Nov 4;15(11):e102124. doi: 10.1136/bmjopen-2025-102124 (PMC12587952; doi:10.1136/bmjopen-2025-102124)

SUPPLEMENTARY MATERIALS

Supplementary Table 1: CHEERS (2022) Checklist

Item	Topic	Page	Section
	**Title**		
1	Title	1	[Title]
	**Abstract**		
2	Abstract	1	Abstract
	**Introduction**		
3	Background and objectives	2	Introduction
	**Methods**		
4	Health economic analysis plan	4	Methods for the economic
5	Study population	3	Trial overview
6	Setting and location	3	Trial overview
7	Comparators	3	Trial overview
8	Perspective	4	Methods for the economic
9	Time horizon	4	Data collection
10	Discount rate	4	Data collection
11	Selection of outcomes	5	Estimating outcomes
12	Measurement of outcomes	5	Estimating outcomes
13	Valuation of outcomes	5	Estimating outcomes
14	Measurement and valuation of resources and costs	5	Estimating costs
15	Currency, price date, and conversion	5	Estimating costs
16	Rationale and description of model	7	Analysis
17	Analytics and assumptions	7	Analysis
18	Characterizing heterogeneity	7	Analysis
19	Characterizing distributional effects	7	Analysis
20	Characterizing uncertainty	7	Analysis
21	Approach to engagement with patients and others affected by the study	8	Patient and public engagement
	**Results**		
22	Study parameters	11	Regression analysis results
23	Summary of main results	11	Regression analysis results
24	Effect of uncertainty	11	Regression analysis results
25	Effect of engagement with patients and others affected by the study	8	Patient and public engagement
	**Discussion**		
26	Study findings, limitations, generalizability, and current knowledge	15	Discussion
	**Other relevant information**		
27	Source of funding	18	Sources of funding
28	Conflicts of interest	19	Conflicts of interest

Supplementary Table 2: Percentage of Patients with Missing Data (Excl. Concomitant Medications)

Variable	Placebo (n=67)	Morphine (n=73)
**Health Outcomes**	*n (%)*	*n (%)*
EQ-5D-5L Health Utility (baseline)	0 (0%)	0 (0%)
EQ-5D-5L Health Utility (day 28)	6 (9%)	9 (12%)
EQ-5D-5L Health Utility (day 56)	7 (10%)	13 (18%)
QALYs (28 days)	6 (9%)	9 (12%)
QALYs (56 days)	8 (12%)	11 (15%)
ICECAP (baseline)	0 (0%)	0 (0%)
ICECAP (day 28)	6 (9%)	9 (12%)
ICECAP (day 56)	7 (10%)	13 (18%)
SF12 6D (baseline)	0 (0%)	0 (0%)
SF12 6D (day 28)	6 (9%)	11 (15%)
SF12 6D (day 56)	7 (10%)	14 (19%)
**Costs (28 days)**	*n (%)*	*n (%)*
Morphine Costs	1 (1%)	1 (1%)
Laxative Costs	1 (1%)	1 (1%)
Inpatient Stay (nights)	6 (9%)	8 (11%)
Emergency Assessment (attendances)	6 (9%)	8 (11%)
Hospital Consultant/Doctor (attendances)	6 (9%)	8 (11%)
Specialist Nurse (attendances)	6 (9%)	8 (11%)
Physiotherapist (attendances)	6 (9%)	8 (11%)
Dietician (attendances)	6 (9%)	8 (11%)
Occupational Therapy (attendances)	6 (9%)	8 (11%)
Social Worker (attendances)	6 (9%)	8 (11%)
Other Outpatient (attendances)	6 (9%)	8 (11%)
Hospital Consultant/Doctor (phone calls)	6 (9%)	8 (11%)
Specialist Nurse (phone calls)	6 (9%)	8 (11%)
Administrator/Secretary (phone calls)	6 (9%)	8 (11%)
Hospice Inpatient Stay (nights)	6 (9%)	8 (11%)
Hospice Doctor (attendances)	6 (9%)	8 (11%)
Hospice Nurse (attendances)	6 (9%)	8 (11%)
Day Hospice (attendances)	6 (9%)	8 (11%)
Other Professional (attendances)	7 (10%)	8 (11%)
Hospice Consultant/Doctor (phone calls)	6 (9%)	8 (11%)
Hospice Specialist Nurse (phone calls)	6 (9%)	8 (11%)
Hospice Administrator/Secretary (phone calls)	6 (9%)	8 (11%)
**Costs (56 days)**	*n (%)*	*n (%)*
Morphine Costs	1 (1%)	1 (1%)
Laxative Costs	1 (1%)	1 (1%)
Inpatient Stay (nights)	9 (13%)	13 (18%)

Variable	Placebo (n=67)	Morphine (n=73)
Emergency Assessment (attendances)	9 (13%)	13 (18%)
Hospital Consultant/Doctor (attendances)	9 (13%)	13 (18%)
Specialist Nurse (attendances)	9 (13%)	13 (18%)
Physiotherapist (attendances)	9 (13%)	13 (18%)
Dietician (attendances)	9 (13%)	13 (18%)
Occupational Therapy (attendances)	9 (13%)	13 (18%)
Social Worker (attendances)	9 (13%)	13 (18%)
Other Outpatient (attendances)	9 (13%)	13 (18%)
Hospital Consultant/Doctor (phone calls)	9 (13%)	13 (18%)
Specialist Nurse (phone calls)	9 (13%)	13 (18%)
Administrator/Secretary (phone calls)	9 (13%)	13 (18%)
Hospice Inpatient Stay (nights)	9 (13%)	13 (18%)
Hospice Doctor (attendances)	9 (13%)	13 (18%)
Hospice Nurse (attendances)	9 (13%)	13 (18%)
Day Hospice (attendances)	9 (13%)	13 (18%)
Other Professional (attendances)	10 (15%)	13 (18%)
Hospice Consultant/Doctor (phone calls)	9 (13%)	13 (18%)
Hospice Specialist Nurse (phone calls)	9 (13%)	13 (18%)
Hospice Administrator/Secretary (phone calls)	9 (13%)	13 (18%)

Supplementary Table 3: Unique Concomitant Medication Entries by Category

Category	Total Entries		Receiving Patients, n (%)	
	Placebo (n=67)	Morphine (n=73)	Placebo (n=67)	Morphine (n=73)
Analgesic	64	78	38 (57%)	43 (59%)
Antidepressant	9	9	9 (13%)	9 (12%)
Antiemetic	3	5	3 (4%)	5 (7%)
Anxiolytic	9	6	8 (12%)	6 (8%)
Corticosteroid	44	50	24 (36%)	31 (42%)
Inhaler	44	52	33 (49%)	37 (51%)
Laxative	12	30	8 (12%)	22 (30%)
Other (excluded)	640	709	67 (100%)	73 (100%)

‘Other’ consists of concomitant medication categories judged as not relevant to the trial intervention and excluded from cost-effectiveness analysis; ‘total entries’ refers to the number of times a drug within a given category has been recorded in the concomitant medications data; ‘receiving patients’ refers to the number (and share) of patients who have received a given a drug within a given category during the study period.

Supplementary Figure 1: MABEL CONSORT Diagram

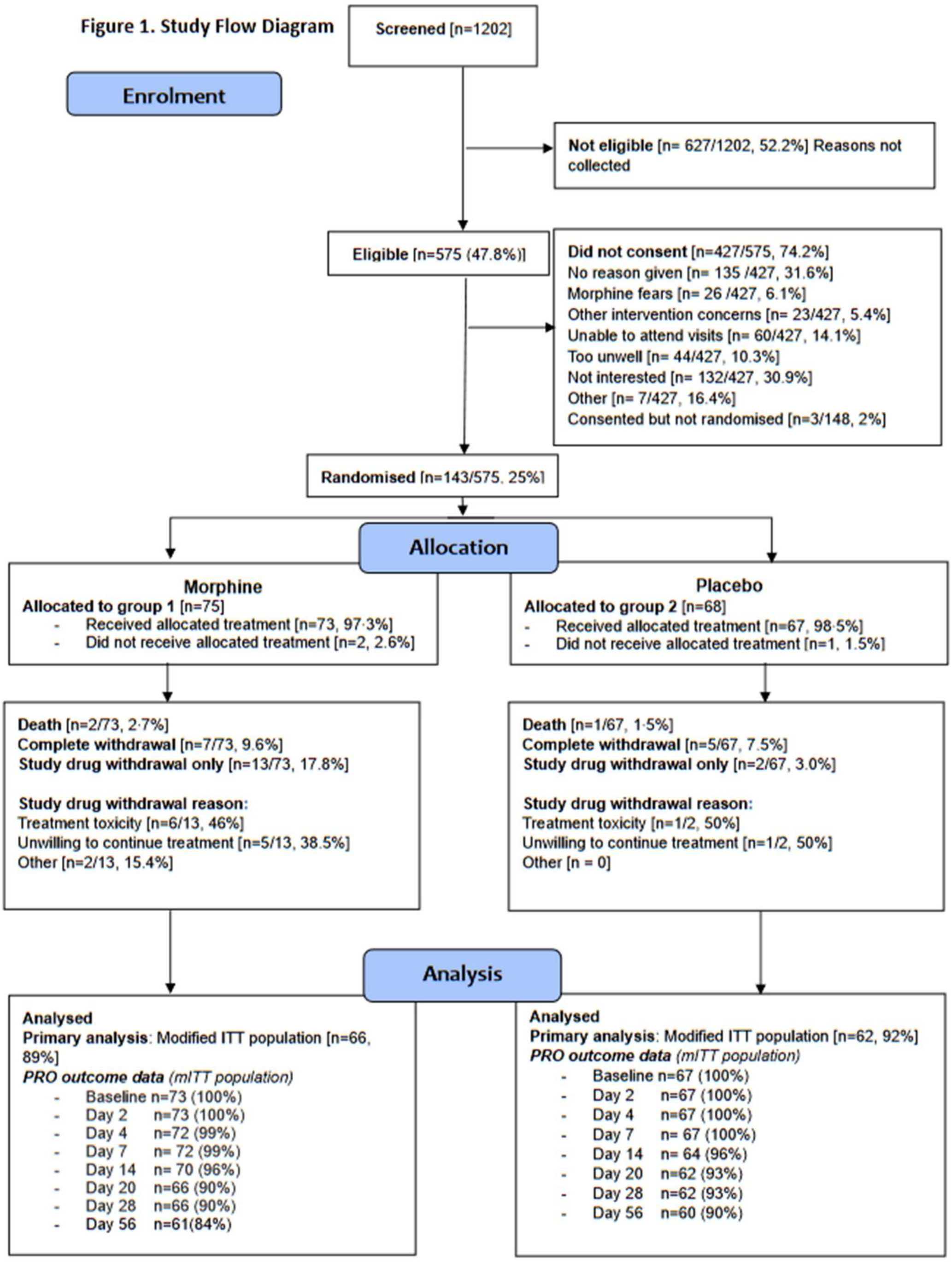

Supplement: online supplemental file 2 [file bmjopen-15-11-s002.pdf]
