# Supplementary figures and images for: Morphine for chronic breathlessness (MABEL) in the UK: a health economic evaluation of a multisite, parallel-group, dose titration, double-blind, randomised, placebo-controlled trial
Source: BMJ Open. 2025 Nov 4;15(11):e102124. doi: 10.1136/bmjopen-2025-102124 (PMC12587952; doi:10.1136/bmjopen-2025-102124)

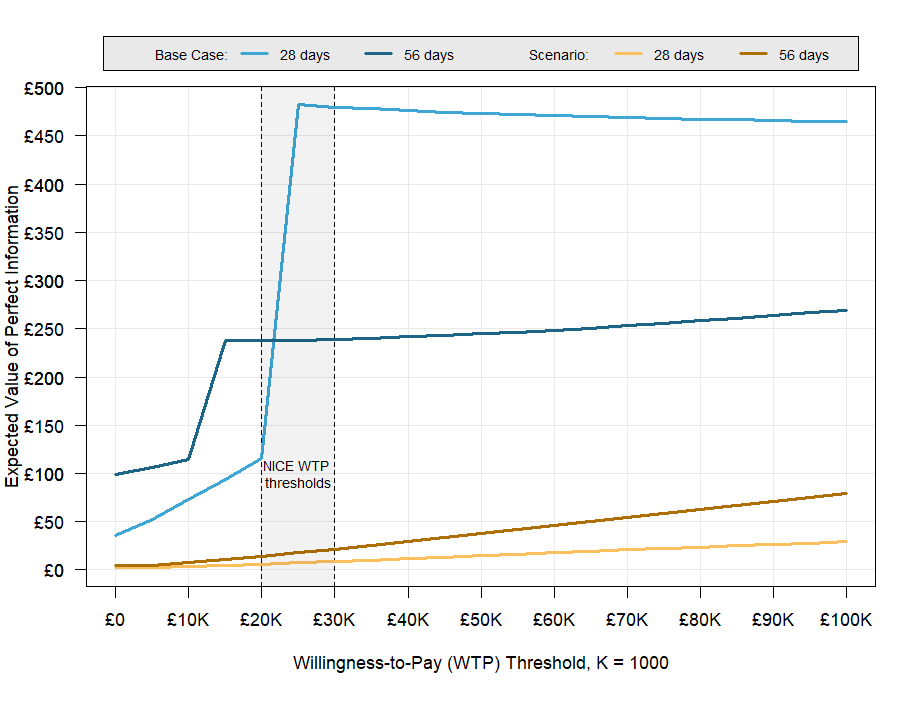

Supplement: online supplemental file 4 [file bmjopen-15-11-s004.png]
