# Supplementary material for: Morphine for chronic breathlessness (MABEL) in the UK: a health economic evaluation of a multisite, parallel-group, dose titration, double-blind, randomised, placebo-controlled trial
Source: BMJ Open. 2025 Nov 4;15(11):e102124. doi: 10.1136/bmjopen-2025-102124 (PMC12587952; doi:10.1136/bmjopen-2025-102124)
Supplement: Supplementary file 5 [file bmjopen-2025-102124.reviewer_comments.pdf]

PEER REVIEW HISTORY

BMJ Open publishes all reviews undertaken for accepted manuscripts. Reviewers are asked to complete a checklist review form and are provided with free text boxes to elaborate on their assessment. These free text comments are reproduced below.

ARTICLE DETAILS

Title (Provisional)

Morphine for chronic breathlessness (MABEL) in the UK: a health economic evaluation of a multi-site, parallel-group, dose titration, double-blind, randomised, placebo-controlled trial

Authors

Atter, Marek Jan; Hall, Peter; Evans , Rachael A; Norrie, John; Cohen, Judith; Williams, Bronwen; Chaudhuri, Nazia; Bajwah, Sabrina; Higginson, Irene; Pearson, Mark; Currow, David; Stewart, Gareth; Fallon, Marie; Johnson, Miriam

---

VERSION 1 - REVIEW

**Reviewer**	**1**
**Name**	**Huot, Laure**
**Affiliation**	**Hospices Civils de Lyon, Pole Sante Publique**
**Date**	**18-Apr-2025**
**COI**	**None**

Comments on “Health economic evaluation of a double-blind, randomized, placebo-controlled trial of low-dose oral morphine (MABEL)”.

Thank you for the opportunity to review this work, that relate a cost consequence analysis based on a randomized controlled trial in moderate to severe breathlessness patients treated with low-dose oral morphine vs. placebo.

The health economic evaluation has been done as part of the MABAL study, and my first major comment concerns the whole trial and the way it is referred to.

Reference 8 appears to report the methods and clinical results of this trial, is not available and is listed as “unpublished manuscript”. Has the manuscript been submitted to another journal? It should be published before mentioning it, or refer to another source (e.g. the protocol registered on ISRCT).

Information on the general methodology of the study is important for the reader's understanding. In all cases, the authors should add the link to the clinical protocol with the ISRCT registration number (methods section). Some more information about the trial

methods should also be added for clearer understanding: precise that the medication strategy was compared to placebo (this only appears in the title of the manuscript); and discuss how this may influence the economic study.

What type of laxative was administered to the patients? Was there also a placebo for this medication in the control group?

The sample size calculation is not presented, so we do not know whether the study was completed in accordance with the planned protocol.

The abstract section refers to the "modified ITT" population, although this is not mention in the methods section: line 25 page 5, "QALYs were calculated… in the ITT population". This has to be clarified, and the definition of mITT (that appears also in the Supplementary Figure 1) should be described.

Unless I a mistaken, the ethics committee decision is not mentioned in the text (number, date of approval) and should be added.

The methodology of the health economic study is in line with standards, even if the societal perspective could have been considered rather than that of the payer.

The cost-consequence analysis has been presented first, and despite the absence of significant difference in costs and QALYs, the cost-utility ratio was calculated. Therefore, results have to be taken with caution, and the authors have correctly addressed this point in their discussion and should highlight it in their conclusions.

Concerning the cost calculation, how were concomitant treatments managed in the control group? Could the placebo have had side effects on the patients, and if so, how did the authors deal with it?

The presentation of results needs to be revised for clarification purposes.

Table 2 contains many calculation and presentation errors! Percentages need to be verified: "white" is not 66% but 94%; % of each primary diagnosis are wrong; page 9 lines 16-17 why 100%?

There are typo errors: decide if you mention % or not in brackets; mean figures are presented with one or two digits after the decimal point. Figures seems to be missing page 9 lines 22-27?

In a more problematic way, it is not clear whether the mean or the median is provided when presenting continuous variables.

Lines from 28 to 39 page 9 precise (SD) but this is not consistent with the rest of the table.

In the table 3 and in the text referring to it, it is not clear if "n" relates to patients or nights/consultations, and based on this, how the means were calculated?

Page 11 line 21, why mention 0/3 SAEs in the placebo group?

Page 11 line 31-32 should be added in in table 4 notes rather than in the text.

The morphine cost at 28 days was £2 per patient: compliance was then 100% for all patients?

Page 15 lines 34-44, the authors discuss about the limitations of performing health economic evaluation as part of the clinical trial. This is not limited to this particular study: cost-effectiveness analysis must be planned at the same time as the clinical protocol, and special attention must be paid to the design of the CRF.

The authors should add discussion of the importance of concomitant treatments in the health economic analysis. Is there some literature about how this may impact on results, according to such population / pathology?

Finally, the authors mention the net health gain form NHS budget: was a budget impact analysis performed or planned within this work?

Two minor comments that may be corrected:

I have a suggestion concerning the title of the manuscript, which should add the population definition.

The titles of additional figures are missing, which makes reading difficult. Supplementary table 2 should mention number of patients.

**Reviewer**	**2**
**Name**	**Wagner, Adam**
**Affiliation**	**NIHR Applied Research Collaboration (ARC) East of England (EoE)**
**Date**	**14-Jun-2025**
**COI**	**None**

Thank you for the opportunity to review this interesting and well written article. I'm particularly impressed with how the authors form the narrative from the diverse results and the sensible conclusions.

Broadly, my feedback is minor, but I would note that certain elements of the HEAP have not been addressed – or, at least, not reported on here – in particular:

• A societal perspective is not explored – which, given the findings of the trial, may add additionally to your narrative

• The HEAP notes QALYs will be calculated from the alternative utility measures, while this is not done in the manuscript.

There may be reasons for not doing the first of these, but I think this should be addressed in the manuscript – to be explicit, please report this or explain why it has not been done. The

latter, if results are not particularly different from the EQ-5D-5L based QALYs, could be included in the Supplementary materials (you note: "SF-6D and ICECAP-SCM scores were not used in QALY calculation or the cost-effectiveness analysis" – but why not when you say in your HEAP you will?).

I try to separate my feedback into what I consider more substantive points, and more minor ones.

Questions:

• Your background notes that much work in this area has focused on COPD – I appreciate the sample size issues, but would a post-hoc subgroup analysis of COPD only participants of the base case be useful for comparing with other studies?

Minor

• I think you should include details of ethical approval in the Trial details section (hence, "No" selected in the "Research ethics" tick box question.

• The modified ITT, compared to ITT, I do not think is explicitly explained in the methods – please add a sentence or two to clarify this (perhaps covered in the main trial paper, but I think this should be covered here for completeness).

• In the background, please add brief detail to motivate the laxative that is included with the IMP (it took until the discussion to appreciate why the intervention included this)

• "Methods for health economic analysis": "In light of the evolving discussion in the scientific literature about the interpretation of the QALY in endof-life settings," – please briefly add detail to cover what these issues are – is it about the sensitivity of these measures? The domains covered? Also, given you comment on *QALYs* here, it would seem most apt to calculate the QALYs from these other measures, as I read your HEAP to imply you were intending to do (linked with the comment above).

• P8: Please could we have some more detail about the web-scraping algorithm? Would you be willing to share your code (supplementary materials?) At least, please could you describe the package you used? This could be useful learning for other health economists

• Table 2: I think needs some refinement – I did not always find it obvious when you switched from reporting means/SD to other summary measures, and/or categorical etc. For example, I think Ethnicity should include %? Also, I think "Placebo" and " Morphine" columns should be swapped to match the other tables. In some places decimal places are not reported consistently (e.g. Pulse oximetry). Spaces are sometimes missing between first number and number in brackets e.g. Cancer row – "1(100)" -> "1 (100)".

• Please add the raw cost-effectiveness planes to the Supplementary materials – I would find this useful to see if there are patterns in the bootstraps etc.

• A value of information analysis is introduced at the end of the results – this is not covered in the methods, nor commented on in the Discussion – please add detail and implication.

Very minor

• Abstract, participants: “Those eligible to participate were” could be removed with no loss.

• Page 4, L35-36: “(measured by a 0-10 numerical rating scale (NRS) on Day 28.” – Close bracket missing before full-stop – perhaps use square backets for outer most brackets?

• You have your base case, and one alternative scenario you explore – in places you refer to this as sensitivity analysis, and others scenario analysis – I appreciate some might consider them interchangeable, but I think best if you adopt standard language throughout.

• Please add the cost year to Table 1 caption, or the footer, to make the table more stand alone. Similarly for other tables where costs are reported.

• P7, L55 – other bullets start with a capital letter

• Page 8, L7 – I’d find it useful if you explained “not relevant” – perhaps in brackets, link to the bullet points above?

• L31/32: “adjusted by the regression controls.” I find this wording odd – could you delete and edit the earlier part of the sentence? “… presents adjusted regression estimates”? In any case, I don’t think “regression controls” is standard language.

• Supplementary materials, Table 3: For consistent formatting, I’d suggest the first letter of each row is Capitalised.

VERSION 1 - AUTHOR RESPONSE

Reviewer: 1

Dr. Laure Huot, Hospices Civils de Lyon, Université Lyon 1

Thank you for the opportunity to review this work, that relate a cost consequence analysis based on a randomized controlled trial in moderate to severe breathlessness patients treated with low-dose oral morphine vs. placebo.

1. The health economic evaluation has been done as part of the MABAL study, and my first major comment concerns the whole trial and the way it is referred to. Reference 8 appears to report the methods and clinical results of this trial, is not available and is listed as “unpublished manuscript”. Has the manuscript been submitted to another journal? It should be published before mentioning it, or refer to another source (e.g. the protocol registered on ISRCT). Information on the general methodology of the study is important for the reader's understanding. In all cases, the authors should add the link to the clinical protocol with the ISRCT registration number (methods section). Some more information about the trial methods should also be added for clearer understanding: precise that the medication strategy was compared to placebo (this only appears in the title of the manuscript); and discuss how this may influence the economic study. What type of laxative was administered to the patients? Was there also a placebo for this medication in the control group?
    a. Thank you for your comment. We agree that detail on trial methodology was lacking, so we have linked the trial protocol/ISRCTN page, expanded on the medication &

placebo strategy, and added a sentence on the economic implications in the *Trial overview* section. The MABEL study has been accepted for publication and is in press in the Lancet Respiratory Medicine journal and has an early DOI (https://doi.org/10.1016/S2213-2600(25)00205-X), so we will update the full reference

2. The sample size calculation is not presented, so we do not know whether the study was completed in accordance with the planned protocol.
    a. We have added this intervention in the *Trial overview* section.
3. The abstract section refers to the "modified ITT" population, although this is not mention in the methods section: line 25 page 5, "QALYs were calculated… in the ITT population". This has to be clarified, and the definition of mITT (that appears also in the Supplementary Figure 1) should be described.
    a. We have clarified this issue in the introduction
4. Unless I a mistaken, the ethics committee decision is not mentioned in the text (number, date of approval) and should be added.
    a. We have added this intervention in the *Trial overview* section.
5. The methodology of the health economic study is in line with standards, even if the societal perspective could have been considered rather than that of the payer.
    a. We have added an explanation for missing items in the HEAP, including this and alternative QALYs. Essentially, this was due to pressure on project timelines and a decision to focus reporting on the payer perspective, which aligns with the standard UK guidance issued by NICE as the national Health Technology Assessment organisation..
6. The cost-consequence analysis has been presented first, and despite the absence of significant difference in costs and QALYs, the cost-utility ratio was calculated. Therefore, results have to be taken with caution, and the authors have correctly addressed this point in their discussion and should highlight it in their conclusions.
    a. Noted, small edit to conclusion to reflect the uncertainty.
7. Concerning the cost calculation, how were concomitant treatments managed in the control group? Could the placebo have had side effects on the patients, and if so, how did the authors deal with it?
    a. Concomitant medication costs were handled identically between arms. Attribution of causality between IMP/placebo was not considered when assigning costs to concomitant medications. We have clarified this in the discussion.
8. The presentation of results needs to be revised for clarification purposes. Table 2 contains many calculation and presentation errors! Percentages need to be verified: "white" is not 66% but 94%; % of each primary diagnosis are wrong; page 9 lines 16-17 why 100%? There are typo errors: decide if you mention % or not in brackets; mean figures are presented with one or two digits after the decimal point. Figures seems to be missing page 9 lines 22-27? In a more problematic way, it is not clear whether the mean or the median is provided when presenting continuous variables. Lines from 28 to 39 page 9 precise (SD) but this is not consistent with the rest of the table.
    a. We have checked and updated Table 2 with these suggestions and marked medians and means separately.
9. In the table 3 and in the text referring to it, it is not clear if "n" relates to patients or nights/consultations, and based on this, how the means were calculated?

    a. We have clarified this in the caption.

10. Page 11 line 21, why mention 0/3 SAEs in the placebo group?
    a. We phrased it this way to highlight that there were 3 SAEs in the Placebo arm base case, but none of them were classed as causal for the purposes of scenario analysis – We have added a short note for clarification.
11. Page 11 line 31-32 should be added in in table 4 notes rather than in the text.
    a. We have moved this to the notes accordingly.
12. The morphine cost at 28 days was £2 per patient: compliance was then 100% for all patients?
    a. We report adherence in the main trial paper which will state that Morphine was well-tolerated with ≥90% Day 28 adherence by 60/73 (82%) participants versus 56/67 (84%) in the placebo group. The £2 is a result of rounding from £1.87 for consistent presentation of cost results.
13. Page 15 lines 34-44, the authors discuss about the limitations of performing health economic evaluation as part of the clinical trial. This is not limited to this particular study: cost-effectiveness analysis must be planned at the same time as the clinical protocol, and special attention must be paid to the design of the CRF.
    a. We respectfully disagree with this point. On page 15, lines 34-44, we are specifically referring to the limitations of collecting free-text concomitant medication data for within-trial analyses in disease areas with high concomitant medication usage, such as MABEL. We do think this section is particularly relevant to this study and offers useful recommendations for similar studies in the future.
14. The authors should add discussion of the importance of concomitant treatments in the health economic analysis. Is there some literature about how this may impact on results, according to such population / pathology?
    a. Yes, we found two relevant papers (DOI: 10.1016/j.jval.2017.02.001, doi: 10.1371/journal.pone.0226868) and expanded on their relevance to our study in the discussion, as well as on the impact of CM on costs and cost-effectiveness.
15. Finally, the authors mention the net health gain form NHS budget: was a budget impact analysis performed or planned within this work?
    a. We did not perform a budget impact as this was outside the study scope.

Two minor comments that may be corrected:

1. I have a suggestion concerning the title of the manuscript, which should add the population definition.
    a. We have now included the population in the title.
2. The titles of additional figures are missing, which makes reading difficult. Supplementary table 2 should mention number of patients.
    a. We think this may just be an issue with the proof PDF, as we have entered figure titles in the relevant field during the submission process. We have added patient numbers to Suppl Table 2.

Reviewer: 2

Dr. Adam Wagner, NIHR Applied Research Collaboration (ARC) East of England (EoE), University of East Anglia

Comments to the Author:

Thank you for the opportunity to review this interesting and well written article. I'm particularly impressed with how the authors form the narrative from the diverse results and the sensible conclusions. Broadly, my feedback is minor, but I would note that certain elements of the HEAP have not been addressed – or, at least, not reported on here – in particular:

1. A societal perspective is not explored – which, given the findings of the trial, may add additionally to your narrative. The HEAP notes QALYs will be calculated from the alternative utility measures, while this is not done in the manuscript. There may be reasons for not doing the first of these, but I think this should be addressed in the manuscript – to be explicit, please report this or explain why it has not been done. The latter, if results are not particularly different from the EQ-5D-5L based QALYs, could be included in the Supplementary materials (you note: "SF-6D and ICECAP-SCM scores were not used in QALY calculation or the cost-effectiveness analysis" – but why not when you say in your HEAP you will?). I try to separate my feedback into what I consider more substantive points, and more minor ones.

    a. We have added an explanation for missing items in the HEAP, including this and alternative QALYs. Essentially, this was due to project timelines/feasibility as we ran out of resources and time to complete this prior to the end of the post for the health economic analyst (MA) - this was due to the need to request an extension to the project due to COVID-19 delays in trial recruitment. We focussed our health economic resource on reporting on the payer perspective, which aligns with the standard UK guidance issued by NICE as the national Health Technology Assessment organisation and we felt was the priority. If resource allows, we would like to report a societal perspective analysis and report this in a future publication.

2. Your background notes that much work in this area has focused on COPD – I appreciate the sample size issues, but would a post-hoc subgroup analysis of COPD only participants of the base case be useful for comparing with other studies?

    a. We have added an acknowledgement that this would have been useful in the limitations and recommended that future research include this. It would be difficult for me to find the time to add a subgroup analysis, as I am no longer working for the trials unit.

Minor

1. I think you should include details of ethical approval in the Trial details section (hence, "No" selected in the "Research ethics" tick box question.

    a. Agreed and added to the trial overview.

2. The modified ITT, compared to ITT, I do not think is explicitly explained in the methods – please add a sentence or two to clarify this (perhaps covered in the main trial paper, but I think this should be covered here for completeness).

    a. We have now addressed and clarified this in the methods.

3. In the background, please add brief detail to motivate the laxative that is included with the IMP (it took until the discussion to appreciate why the intervention included this)

    a. Agreed and added to trial overview.

4. "Methods for health economic analysis": "In light of the evolving discussion in the scientific literature about the interpretation of the QALY in endof-life settings," – please briefly add detail to cover what these issues are – is it about the sensitivity of these measures? The domains covered? Also, given you comment on *QALYs* here, it would seem most apt to calculate the QALYs from these other measures, as I read your HEAP to imply you were intending to do (linked with the comment above).

    a. We have added a short summary of justification for presenting the alternative measures. The lack of alternative QALYs is covered in our response to the previous comment.

5. P8: Please could we have some more detail about the web-scraping algorithm? Would you be willing to share your code (supplementary materials?) At least, please could you describe the package you used? This could be useful learning for other health economists
    a. We cannot share the full code as it no longer works. At the time of analysis (Summer 2024), the BNF website accepted web-scraping requests. However, during the resubmission process, the code stopped working due to a 403 access denial error, which we found to be due to a change in the BNF's end-user licensing agreement forbidding web-scraping. Instead, we have referenced the "rvest" package documentation we used – this may health economists scrape cost data in other databases.
6. Table 2: I think needs some refinement – I did not always find it obvious when you switched from reporting means/SD to other summary measures, and/or categorical etc. For example, I think Ethnicity should include %? Also, I think "Placebo" and " Morphine" columns should be swapped to match the other tables. In some places decimal places are not reported consistently (e.g. Pulse oximetry). Spaces are sometimes missing between first number and number in brackets e.g. Cancer row – "1(100)" -> "1 (100)".

    a. Noted, apologies for the mistakes in this section. We have checked and corrected/reformatted Table 2.
7. Please add the raw cost-effectiveness planes to the Supplementary materials – I would find this useful to see if there are patterns in the bootstraps etc.

    a. We have reworked the CEPs. We previously chose not to present them raw due to readability/comparison issues, but we have now found an improved way to visualise them and add Day 28 CEPs as well. For transparency in terms of Bootstrap patterns, we have also supplied CEPs with costs plotted on a log scale in the supplementary materials and explained our justification for doing so.
8. A value of information analysis is introduced at the end of the results – this is not covered in the methods, nor commented on in the Discussion – please add detail and implication.

    a. Thank you for raising this, as thanks to this, we noticed that the VoI figure I originally supplied is outdated (based on a previous bootstrap run with incomplete calculations) – we apologise for this error. We have now submitted a corrected VoI figure and expanded on it in the methods and discussions section.

Very minor

1. Abstract, participants: “Those eligible to participate were” could be removed with no loss.
    a. Done.
2. Page 4, L35-36: “(measured by a 0-10 numerical rating scale (NRS) on Day 28.” – Close bracket missing before full-stop – perhaps use square backets for outer most brackets?
    a. Done
1. You have your base case, and one alternative scenario you explore – in places you refer to this as sensitivity analysis, and others scenario analysis – I appreciate some might consider them interchangeable, but I think best if you adopt standard language throughout.

    a. We agree with this suggestion and have changed the wording to “scenario”.
2. Please add the cost year to Table 1 caption, or the footer, to make the table more stand alone. Similarly for other tables where costs are reported.

    a. Added to caption.

3. P7, L55 – other bullets start with a capital letter

    a. Fixed.

4. Page 8, L7 – I’d find it useful if you explained “not relevant” – perhaps in brackets, link to the bullet points above?

    a. This refers to concomitant medicines that are considered not to be causally related to the IMP (or placebo) it its side effects. We have added additional explanatory text to the manuscript.

5. L31/32: “adjusted by the regression controls.” I find this wording odd – could you delete and edit the earlier part of the sentence? “… presents adjusted regression estimates”? In any case, I don’t think “regression controls” is standard language.

    a. Agreed, we have changed the wording
6. Supplementary materials, Table 3: For consistent formatting, I’d suggest the first letter of each row is Capitalised.

    a. Done.
